# Task difficulty modulates the effect of mind wandering on phase dynamics

**DOI:** 10.1073/pnas.2416387122

**Published:** 2025-05-30

**Authors:** Zhengkun Long, Georg Northoff, Xiaolan Fu

**Affiliations:** ^a^State Key Laboratory of Cognitive Science and Mental Health, Institute of Psychology, Chinese Academy of Sciences, Beijing 100101, China; ^b^Department of Psychology, University of Chinese Academy of Sciences, Beijing 100049, China; ^c^School of Psychology, Shenzhen University, Shenzhen, Guangdong 518060, China; ^d^Mind, Brain Imaging and Neuroethics Research Unit, The Royal’s Institute of Mental Health Research, University of Ottawa, Ottawa, ON K1Z 7K4, Canada; ^e^School of Psychology, Shanghai Jiao Tong University, Shanghai 200030, China

**Keywords:** mind wandering, task difficulty, phase coherence, temporal precision, internal and external cognition

## Abstract

Mind wandering attenuates widespread sensory and motor processing, both of which are mediated by phase coherence. However, it remains unclear i) whether mind wandering impacts both sensory input and motor output processing by modulating ii) neural entrainment to external stimuli, as measured by intertrial phase coherence (ITPC), and specifically iii) whether task difficulty with different degrees of attentional demands moderates the impact of mind wandering on phase coherence. Using the thought-probe method, we assessed participants’ attentional states during different sensory and motor tasks with varying task difficulty. We found that mind wandering decreased ITPC exclusively in less demanding tasks but not in difficult ones, regardless of whether the tasks involved visual input or motor output processing. Our results suggest that external task difficulty may modulate the balance between external and internal cognitive processing (e.g., mind wandering), with simpler tasks facilitating internally oriented cognition and increasing mind wandering. This balance between internal mind wandering and external task difficulty is mediated, in part, by phase coherence, which serves as an underlying neural mechanism. Collectively, our findings support the hypothesis that phase coherence and its dynamics (ITPC) play a key role in mediating the reciprocal balance of internal and external cognition—this suggests their partly shared cognitive-executive resources as entailed by the recently proposed Baseline model of cognition.

## Significance

We demonstrate that external (sensory and motor processing) and internal cognition (mind wandering) are dynamically interdependent. Easier external tasks require fewer executive resources, leaving more cognitive resources for internal mind wandering. Increased internal mind wandering reduces the brain’s responsiveness to external events, as evidenced by decreased phase coherence (temporal imprecision) compared to an on-task state. Conversely, in difficult tasks, phase coherence between neural activity and external events remains high (indicating temporal precision) and is thus unaffected by mind wandering. This moderating effect of task difficulty on phase coherence underscores the reciprocal balance between internal and external cognition, indicating that they likely share underlying cognitive-executive resources and neurophysiological mechanisms, providing empirical support for the Baseline model of cognition.

Imagine you are playing badminton with a friend, but recent unfinished tasks preoccupy your mind, preventing you from fully tracking the shuttlecock’s flight trajectory. As a result, your attention is constantly diverted, and you struggle to react promptly. This phenomenon, known as mind wandering in academic research, involves attention shifting from the ongoing task to task-unrelated thoughts (1, 2). Mind wandering is worth studying for several reasons. First, it is a significant psychological experience in individuals’ daily lives. Research has found that individuals spend nearly 30% of their time in a state of mind wandering (3–7). Second, mind wandering can impact individuals’ cognition and behavior (8–10). In this sense, mind wandering offers insights into how the mind functions and its relationship with the brain, including the interplay between internal and external cognition.

The perceptual decoupling hypothesis posits that mind wandering is a state where the content of one’s thoughts is not related to the stimulus or task at hand, thereby becoming decoupled from the perception of the external stimulus or task (7, 11). This implies that mind wandering may reduce cognitive processing of external information and redirect attention to internal thoughts. Using electroencephalography (EEG), numerous event-related potential (ERP) studies have strongly supported this hypothesis. The amplitudes of both early sensory processing ERPs (9, 12) and later cognitive processing ERPs (13–16) decrease during mind wandering. Among these components, the P300 is believed to mirror the allocation of executive resources to stimuli (17). The reduction in P300 amplitude during mind wandering suggests that executive and attentional resources are at least partially diverted from the primary task to process task-unrelated thoughts (16). Furthermore, mind wandering also affects motor processing (10, 18, 19). The common modulation of both sensory and motor processing by mind wandering suggests a potentially shared mechanism underlying both sensory and motor attenuation during internal cognition (8). However, the nature of this shared mechanism remains unclear, and our study aims to address this.

Sensory processing exhibits neural entrainment—the synchronization of intrinsic neural oscillations with external rhythmic stimuli at specific time points (20). When considered over extended timescales, this synchronization reflects neural alignment, a broader concept referring to the temporal correspondence between neural activity and external events across multiple timescales (21). Entrainment is measured at specific time points through the periodic fluctuations of neural oscillatory activity, including peak/trough and rise/fall. These fluctuations can be represented by different phase angles (22) (ranging from 0 to 2π), whose timing—e.g., coherence—can synchronize and thus align with the timing of external sensory or motor stimuli. Intertrial phase coherence (ITPC) quantifies the consistency of oscillatory phase angles across trials at specific time-frequency points relative to stimulus onset (20, 23, 24), providing a measure of the temporal precision with which neural responses synchronize with external stimuli. Thus, ITPC serves as a proxy for temporal precision (exact phase timing) and temporal segregation (e.g., distinguishing stimulus onset from other events) relative to the temporal onset of the external input (20, 25). Higher ITPC indicates stronger phase coherence across trials, meaning phase angles are more tightly aligned with stimulus-driven responses, which in turn reflects reduced temporal flexibility. Conversely, lower ITPC reflects greater trial-to-trial phase variability, indicating phase incoherence and temporal imprecision.

More generally, phase coherence reflects the dynamic interaction of the rhythmic activity of the brain’s internal dynamics with the temporal features of the external stimuli (20). If mind wandering (i.e., internal cognition) increases, leading to attenuated processing of the external input, one would expect it also to reduce the brain’s temporal precision relative to the timing of the external stimuli as measured by phase coherence, e.g., ITPC. Specifically, the question arises whether mind wandering, as an internally driven cognitive activity, intermittently corrupts and overwhelms the processing of external stimuli, thereby making neuronal activity less regular and consistent, ultimately diminishing the temporal precision of neural responses to external stimuli, as reflected in ITPC. Investigating this effect may thus shed light on a potential trade-off in the brain’s capacity to allocate and balance cognitive-executive resources between mind wandering/internal cognition and stimulus processing/external cognition.

This potential trade-off between internal and external cognition raises questions about how mind wandering might affect ongoing neural oscillatory dynamics. Does mind wandering affect the brain’s ongoing phase cycles? Indeed, one recent EEG study showed that mind wandering, like on–off thoughts, is related to phase cycles as measured by their phase angles in frequency sliding (26). Another previous study found that mind wandering impairs motor control only during simple movements, with no effect on relatively difficult movements (10). Thus, task difficulty emerges as a crucial modulating factor to consider when examining how internal mind wandering influences external processing. In summary, these findings raise the question of whether mind wandering impacts and possibly reduces the ITPC in response to external sensory and motor tasks and stimuli of varying difficulties. We therefore examine whether phase coherence, e.g., ITPC, provides the link between external cognition involving both sensory and motor stimuli/tasks (including their varying task difficulty) on the one hand and internal cognition with mind wandering on the other—this would support the assumption that internal and external cognition share, in part, their underlying neural substrates (e.g., ITPC) as proposed by the Baseline model of cognition (27).

**Fig. 1.**
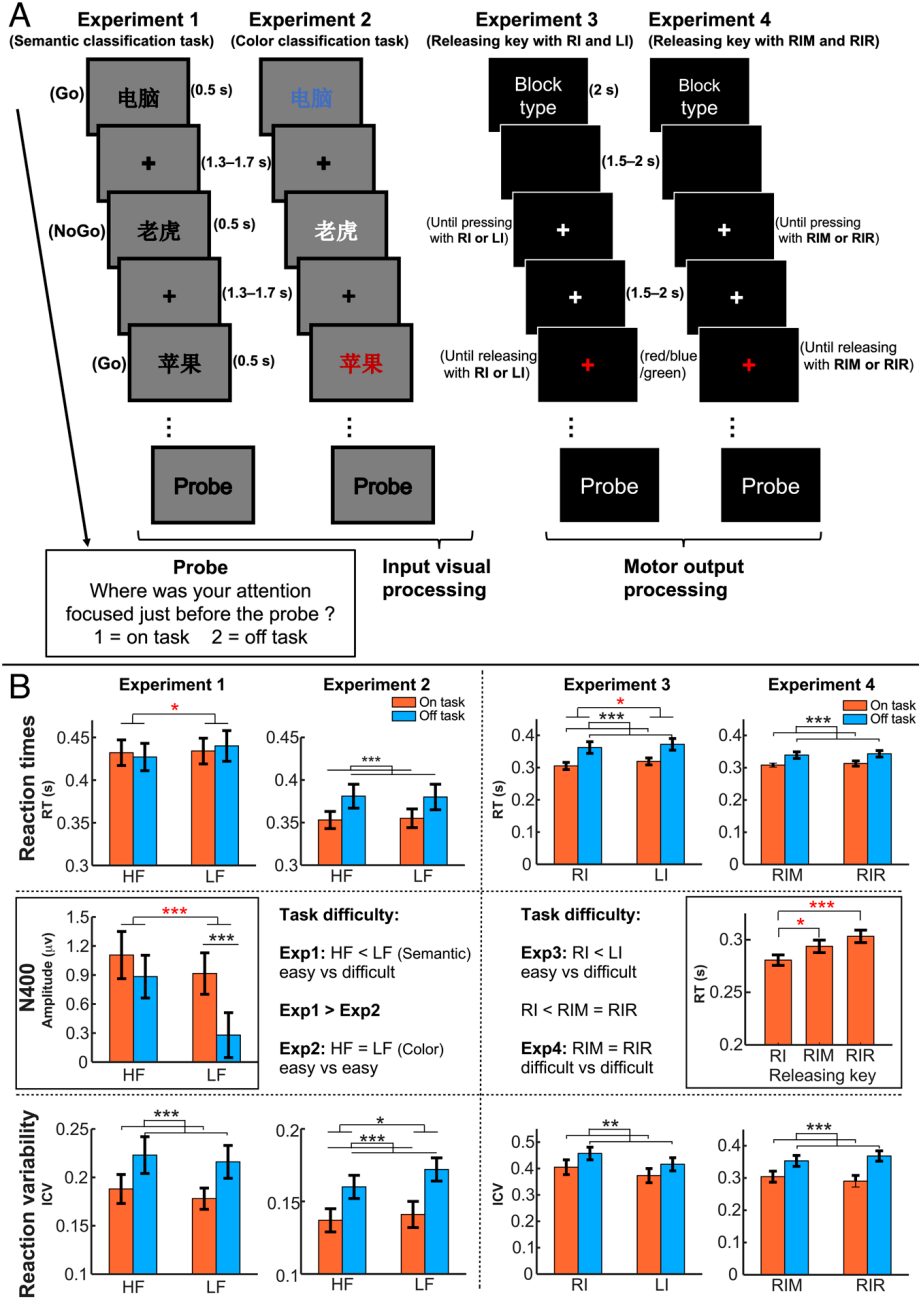
Schematic representation of the experimental tasks and the design of task difficulty with supporting evidence. (*A*) Experiment 1 was a SART with Chinese words, where participants pressed a button for nonanimal (nontarget) words and withheld responses for animal (target) words. Experiment 2 followed the same design but required participants to judge word color, pressing a button for red, blue, green, or black words and withholding responses for white words. Experiment 3 was a motor task where participants quickly pressed the “P” or “Q” key with their right (RI) or left (LI) index finger upon seeing a white fixation cross, holding the key until the cross changed color, then releasing it. Experiment 4 had the same design as Experiment 3 but required simultaneous movements with two right-hand fingers (RIM or RIR). All experiments used a block design with randomized trials, and at the end of each block, participants reported whether they were “on task” or “off task.” (*B*) Experiment 1 manipulated semantic processing difficulty using word frequency, with longer reaction times and a larger N400 for LF words compared to HF words. Thus, the HF condition was considered easy, and the LF condition difficult. Experiment 2 was easier, with overall faster reaction times (compared to Experiment 1) and no difference between HF and LF conditions, classifying both as easy. Experiment 3 manipulated movement difficulty through handedness, showing longer reaction times for LI than RI finger movements, classifying RI as easy and LI as difficult. In Experiment 4, both RIM and RIR finger movements were considered difficult, supported by slower reaction times compared to RI in a preliminary test. All four experiments showed that mind wandering increased behavioral variability (greater ICV). Detailed results are in *SI Appendix*, *Supplementary Results*. *P < 0.05; ***P < 0.001.

We used the thought-probe method (1, 28) in two sensory tasks, e.g., sustained attention to response tasks (SART, Experiments 1 and 2) and two motor tasks, e.g., signal response tasks (Experiments 3 and 4) with varying task difficulty to detect the participants’ attentional states (“on task” or “off task”; see Fig. 1*A*). According to the resource-control account of sustained attention (29), mind wandering and external task processing share the same executive control resources. Easier external tasks, requiring less control and effort, may engage fewer executive resources, leaving more cognitive capacities available for mind wandering. Thus, easier tasks with lower cognitive demands may be more adversely affected by internal mind wandering than more difficult ones. In the current study, task difficulty was manipulated based on the cognitive demands of the task. The most direct indicator of these demands is reaction time: Tasks requiring more cognitive resources, whether sensory or motor, are associated with slower reaction times (30), which we interpret as reflecting higher task difficulty. Although reaction times varied with task difficulty in Experiments 1 and 2 of the SART, the absence of differences in commission errors across conditions (*SI Appendix,* Fig. S3) suggests that the difficulty manipulation did not fundamentally alter the core speed–accuracy trade-off intrinsic to the SART framework (31). This stability confirms the SART’s validity for studying mind wandering, even with variations in difficulty.

Experiment 1 used high- and low-frequency (HF and LF) words to manipulate the semantic processing difficulty, a method derived from a recent study by Long et al. (14). At the left occipital electrodes (P7, PO7, and PO5) associated with word recognition [referencing the left-lateralized N170 (32) and the visual word form area (33)], we anticipated that mind wandering would show decreased phase coherence (ITPC) in the easier HF word condition while not affecting the more difficult LF word condition. Experiment 2 used the same design as Experiment 1 but shifted from judging semantic information to judging color information, a relatively simple task. Therefore, we expected that at the occipital electrodes (left: P7, PO7, PO5; right: P8, PO8, PO6) associated with visual processing (34), mind wandering would reduce the ITPC for both HF and LF word conditions. However, when participants were required to focus on the task-relevant color dimension that requires executive-control processes, the automatically activated task-irrelevant semantic dimension would interfere (35). Theta (4 to 7 Hz) oscillations have been extensively linked to cognitive control processes, with their phase-locked dynamics in midfrontal areas serving as a key neural correlate underlying the integration of choice-relevant information during goal-directed behavior (36–38). In Experiment 2, participants needed to prioritize task-relevant color information over task-irrelevant semantic information. We, therefore, expected that mind wandering would reduce the midfrontal theta (4 to 7 Hz) ITPC for “low” task difficulty, e.g., HF words, but not for “high” task difficulty as in LF words.

Experiments 3 and 4 transitioned from sensory input to motor output processing by manipulating movement difficulty through handedness and finger dexterity. Using experimental paradigms derived from recent work by Long et al. (10), we examined how motor execution is affected by mind wandering. In both experiments, participants performed simple button-press tasks with either dominant or nondominant hands (Experiment 3) and with fingers of varying dexterity (Experiment 4). Our hypothesis is based on Baker et al. (39, 40), who demonstrated that the activity of neural assemblies in the primary motor cortex (M1) undergoes strong modulation during the different phases of a precision grip task. Additionally, Popovych et al. (41) found that phase locking in the delta-theta (2 to 7 Hz) frequency band at contralateral motor regions is a ubiquitous property of motor execution. Through both studies, we expected that mind wandering would reduce delta-theta (2 to 7 Hz) ITPC of relatively simple movements during motor execution but would not influence more difficult movements. Understanding how oscillatory neural dynamics are affected by mind wandering may reveal fundamental principles about how the brain balances internal thought processes and external task demands.

## Results

### Experiment 1: Sensory Cognitive Processing in the Semantic Classification Task.

To examine whether task difficulty modulates the effect of mind wandering on phase coherence during visual input processing, we used HF and LF words to vary semantic difficulty. Reaction times were slower, and N400 and P200 components were larger for LF words, indicating greater semantic difficulty in the LF condition compared to the HF condition (Fig. 1*B* and *SI Appendix,* Fig. S1). Reaction variability [intraindividual coefficient of variation (ICV) of RT] was higher for nontarget words preceding off-task reports across both conditions (Fig. 1*B*). Mind wandering reports averaged ~50% across the experiment, with no significant difference between HF (45.79%, *SE* = 2.27%) and LF conditions (45.36%, *SE* = 2.46%). Since Experiment 1 employed the SART paradigm, we also report behavioral responses corresponding to commission errors (failures to withhold responses to target stimuli) in *SI Appendix,* Fig. S4.

**Fig. 2.**
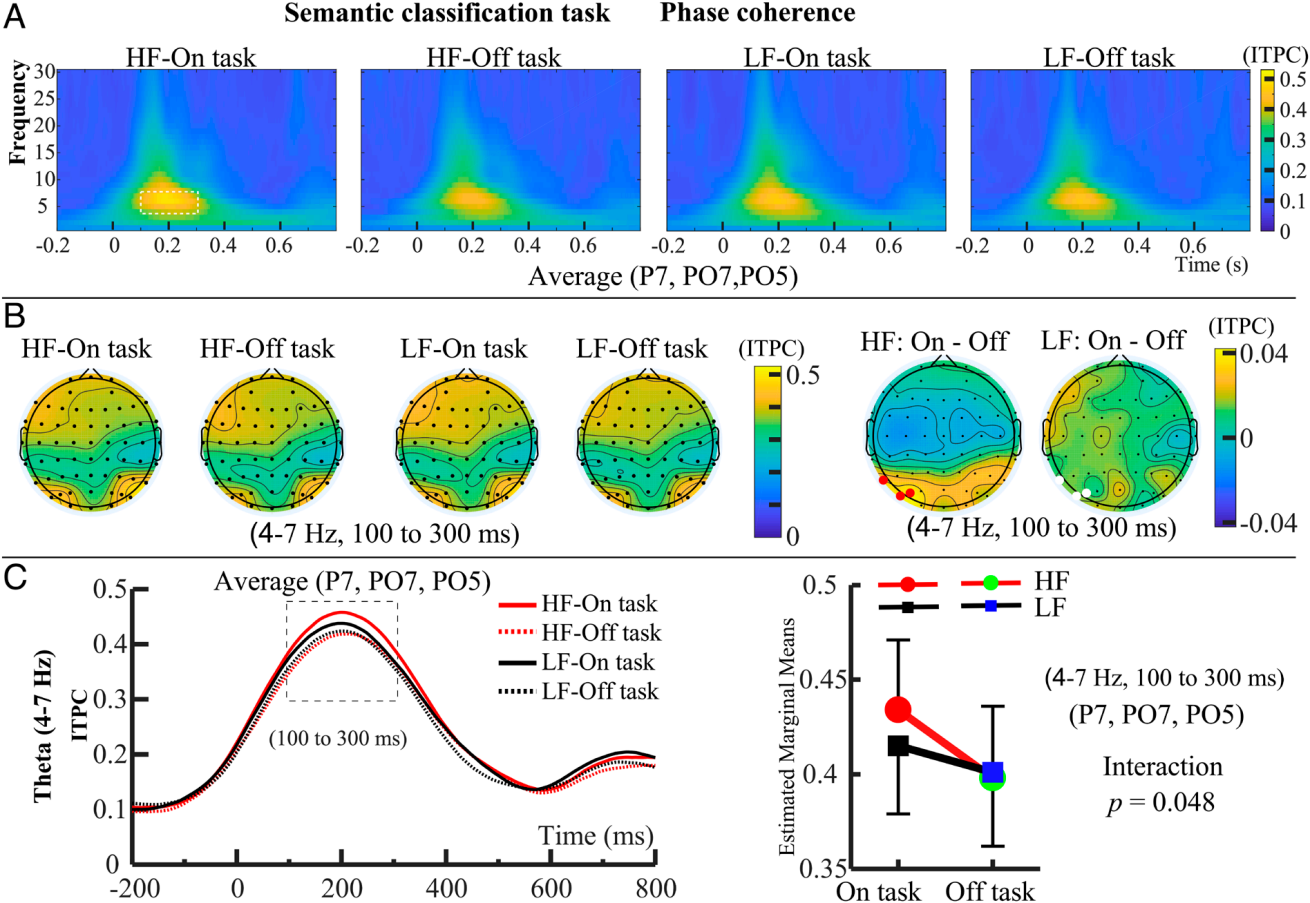
Phase coherence results of Experiment 1, the semantic classification task. (*A*) The *Top* panel shows ITPC results for the left occipital electrodes (P7, PO7, PO5), with phase coherence observed 100 to 300 ms after word presentation, primarily in the theta band (4 to 7 Hz). The ROI was defined as this 100 to 300 ms window in the theta band (white dashed box) for further analysis. (*B*) The *Middle* panel presents topographical maps of theta-band ITPC for each condition within the 100 to 300 ms range, along with the difference topography. Electrodes used for statistical analysis are marked in red and white, with red indicating significant differences between the “on task” and “off task” states. (*C*) For ITPC data from P7, PO7, and PO5 electrodes (100 to 300 ms; 4 to 7 Hz), mind wandering reduced phase coherence only in the HF condition, with no effect on the LF condition.

Since Experiment 1 required participants to perform semantic processing, we selected ITPC data from P7, PO7, and PO5 electrodes in the left occipital area (Fig. 2*B*), which is associated with word recognition (32, 33), for subsequent statistical analysis. The selection of electrodes was also supported by permutation test results (*SI Appendix,* Fig. S5). Since our primary focus was on the impact of mind wandering on the degree of phase coherence in those frequency bands that are known to be related in a specific way to our tasks, we did not examine all frequency bands. Specifically, driven by prior knowledge, we focused on the theta band (4 to 7 Hz) as which is known to support the interface between language and memory (42). This focus aligns with the demands of our task, which required participants to retrieve semantic information from long-term memory to classify word meanings. Phase coherence of visual input processing mainly occurred at theta band (4 to 7 Hz), approximately from 100 to 300 ms after word onset, which was selected as the time period of interest (Fig. 2*A*). We performed a 2 (probe: On task vs. Off task) × 2 (word frequency: HF vs. LF) repeated-measures ANOVA (Fig. 2*C*). It revealed a significant main effect of probe, *F*(1, 28) = 14.368, *P* < 0.001, *η_p_*^2^ = 0.339, and a significant interaction between probe and word frequency, *F*(1, 28) = 4.271, *P* = 0.048, *η_p_*^2^ = 0.132, but no significant main effect of word frequency, *F*(1, 28) = 1.268, *P* = 0.270, *η_p_*^2^ = 0.043. Simple effect analysis revealed that the theta ITPC of HF-Off task was smaller than that of HF-On task (*P* < 0.001, *η_p_*^2^ = 0.359), whereas there was no significant difference between LF-On task and LF-Off task (*P* = 0.657, *η_p_*^2^ = 0.100). Noninferiority *t* tests with a margin of 0.01 were conducted. For the HF condition, the test was significant, *t*(28) = 2.88, *P* = 0.008, 95% CI [0.018, 0.056], indicating that mind wandering reduced theta ITPC. In contrast, the test for the LF condition was not significant, *t*(28) = 0.51, *P* = 0.62, 95% CI [−0.002, 0.030], showing no modulation of theta ITPC by mind wandering during the more difficult task condition.

### Experiment 2: Sensory Processing in the Color Classification Task.

**Fig. 3.**
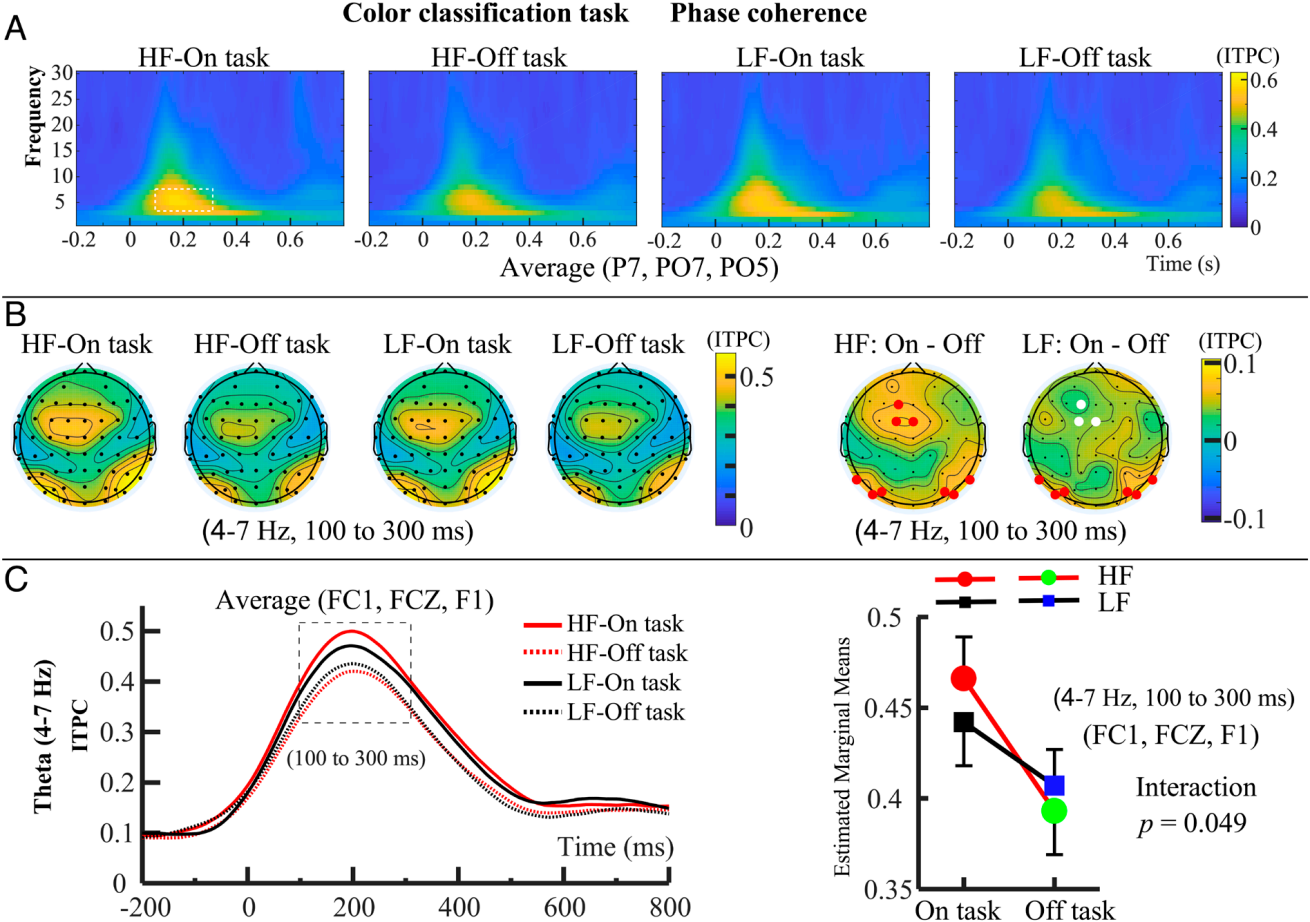
Phase coherence results of Experiment 2, the visual color classification task with the same word stimuli as in Experiment 1. (*A*) Similar to Fig. 2, the *Top* panel shows ITPC results for the left occipital electrodes (P7, PO7, PO5). In subsequent analyses, we focused on the theta band, where phase coherence in visual input processing was most prominent in the 4 to 7 Hz range, centered around 100 to 300 ms after word presentation. This 100 to 300 ms window was selected as the ROI, marked by the white dashed box. (*B*) The *Middle* panel shows topographical maps of theta-band ITPC for each condition within the 100 to 300 ms range, along with the difference topography. Electrodes used for statistical analysis are marked in red and white, with red indicating significant differences between “on task” and “off task” states. (*C*) For ITPC data from FC1, FCZ, and F1 electrodes (100 to 300 ms; 4 to 7 Hz), mind wandering reduced phase coherence only in the HF condition, with no effect on the LF condition.

Experiment 2 investigated the task difficulty in the domain of pure visual processing, namely color judgment rather than word recognition. Mind wandering slowed reaction times during off-task thoughts, indicating its impact on reaction speed in this simple sensory task. While reaction times showed no significant difference between HF and LF words, this suggests a smaller word frequency effect in Experiment 2 compared to Experiment 1 (see also Figs. 2 and 3, ITPC at left occipital electrodes P7, PO7, PO5). Mind wandering also increased reaction time variability, with greater variability for LF words, likely due to stronger semantic interference. This suggests that, despite being task-irrelevant, semantic processing difficulty may still affect task performance (Fig. 1*B* and *SI Appendix*, *Supplementary Results*). Mind wandering reports averaged ~50% across the experiment, with no significant difference between HF (46.55%, *SE* = 1.88%) and LF conditions (45.31%, *SE* = 2.27%) (*SI Appendix,* Fig. S2). Behavioral results show faster reaction times before commission errors than correct inhibitions, with no difference in reaction variability (*SI Appendix,* Fig. S4). Together with Experiment 1, this suggests that commission errors may result from motor decoupling rather than mind wandering, as further discussed in *SI Appendix,* Fig. S4.

Since Experiment 2 required participants to judge simple color information, involving only basic visual processing, we selected ITPC data from occipital electrodes (left: P7, PO7, PO5; right: P8, PO8, PO6) (Fig. 3*B*), which are associated with visual processing (34), for subsequent statistical analysis. Similar to Experiment 1, phase coherence of visual input processing primarily occurred in the theta band (4 to 7 Hz) within the 100 to 300 ms following word presentation (Fig. 3*A*). We selected the average ITPC within the 4 to 7 Hz, 100 to 300 ms time-frequency window as the region of interest (ROI) and performed a 2 (probe: On task vs. Off task) × 2 (word frequency: HF vs. LF) repeated-measures ANOVA. For the left occipital P7, PO7, PO5 electrodes, it revealed a significant main effect of probe, *F*(1, 28) = 6.442, *P* = 0.017, *η_p_*^2^ = 0.187, a marginal significant main effect of word frequency, *F*(1, 28) = 4.155, *P* = 0.051, *η_p_*^2^ = 0.129, but no significant interaction between probe and word frequency, *F*(1, 28) = 0.004, *P* = 0.950, *η_p_*^2^ < 0.001. For the right occipital P8, PO8, PO6 electrodes, it revealed a significant main effect of probe, *F*(1, 28) = 8.206, *P* = 0.008, *η_p_*^2^ = 0.227, but no significant main effect of word frequency, *F*(1, 28) = 0.436, *P* = 0.515, *η_p_*^2^ = 0.015, and no significant interaction between probe and word frequency, *F*(1, 28) = 0.019, *P* = 0.891, *η_p_*^2^ < 0.001. These results indicate that mind wandering reduces phase coherence of visual input processing during easy visual sensory tasks.

Although Experiment 2 instructed participants to focus on color and ignore semantic information, the semantic properties of Chinese words may have been automatically activated, influencing task performance. This is supported by a marginally significant main effect of word frequency at left occipital electrodes (P7, PO7, PO5), associated with semantic processing, consistent with electrode selection in Experiment 1. Participants were required to prioritize task-relevant color information over task-irrelevant semantic information. To examine the influence of task-irrelevant semantic activation, we analyzed midfrontal theta ITPC (FC1, FCZ, F1 electrodes; see Fig. 3*C*), which plays a key role in integrating choice-relevant information during goal-directed behavior, such as when participants must select one response over another (36–38). It revealed a significant main effect of probe, *F*(1, 28) = 8.171, *P* = 0.008, *η_p_*^2^ = 0.226, and a significant interaction between probe and word frequency, *F*(1, 28) = 4.211, *P* = 0.049, *η_p_*^2^ = 0.131, but no significant main effect of word frequency, *F*(1, 28) = 0.463, *P* = 0.502, *η_p_*^2^ = 0.016. Simple effect analysis revealed that the midfrontal theta ITPC of HF-Off task was smaller than that of HF-On task (*P* = 0.007, *η_p_*^2^ = 0.296) while there was no significant difference between LF-On task and LF-Off task (*P* = 0.626, *η_p_*^2^ = 0.092). Noninferiority *t* tests with a margin of 0.01 were conducted. For the HF condition, the test was significant, *t*(28) = 2.92, *P* = 0.006, 95% CI [0.029, 0.117], indicating that mind wandering significantly reduced midfrontal theta ITPC. While for the LF condition, the test was not significant, *t*(28) = 1.20, *P* = 0.240, 95% CI [−0.008, 0.078], indicating no significant effect of mind wandering on midfrontal theta ITPC. These results suggest that mind wandering reduced phase coherence in the processing of task-irrelevant visual information during the relatively easy task (“HF”). In contrast, this effect was not observed in the more difficult task condition (“LF”).

### Experiment 3: Motor Processing in the Motor Task of Key-Releasing with RI and LI.

Experiment 3 examined whether task difficulty modulates the impact of mind wandering on motor output processing by manipulating movement difficulty through handedness. Right-handed participants showed faster reaction times with the right index (RI) finger than the left index (LI) finger, indicating that RI key-releasing was easier (Fig. 1*B*). Reaction time variability (ICV of RT) was higher before “off-task” than “on-task” reports for both RI and LI conditions, reflecting more variable responses during mind wandering (Fig. 1*B*). Mind wandering reports averaged ~50%, with no significant difference between RI (50.73%, SE = 1.46%) and LI (49.83%, SE = 2.12%) conditions (*SI Appendix,* Fig. S2).

**Fig. 4.**
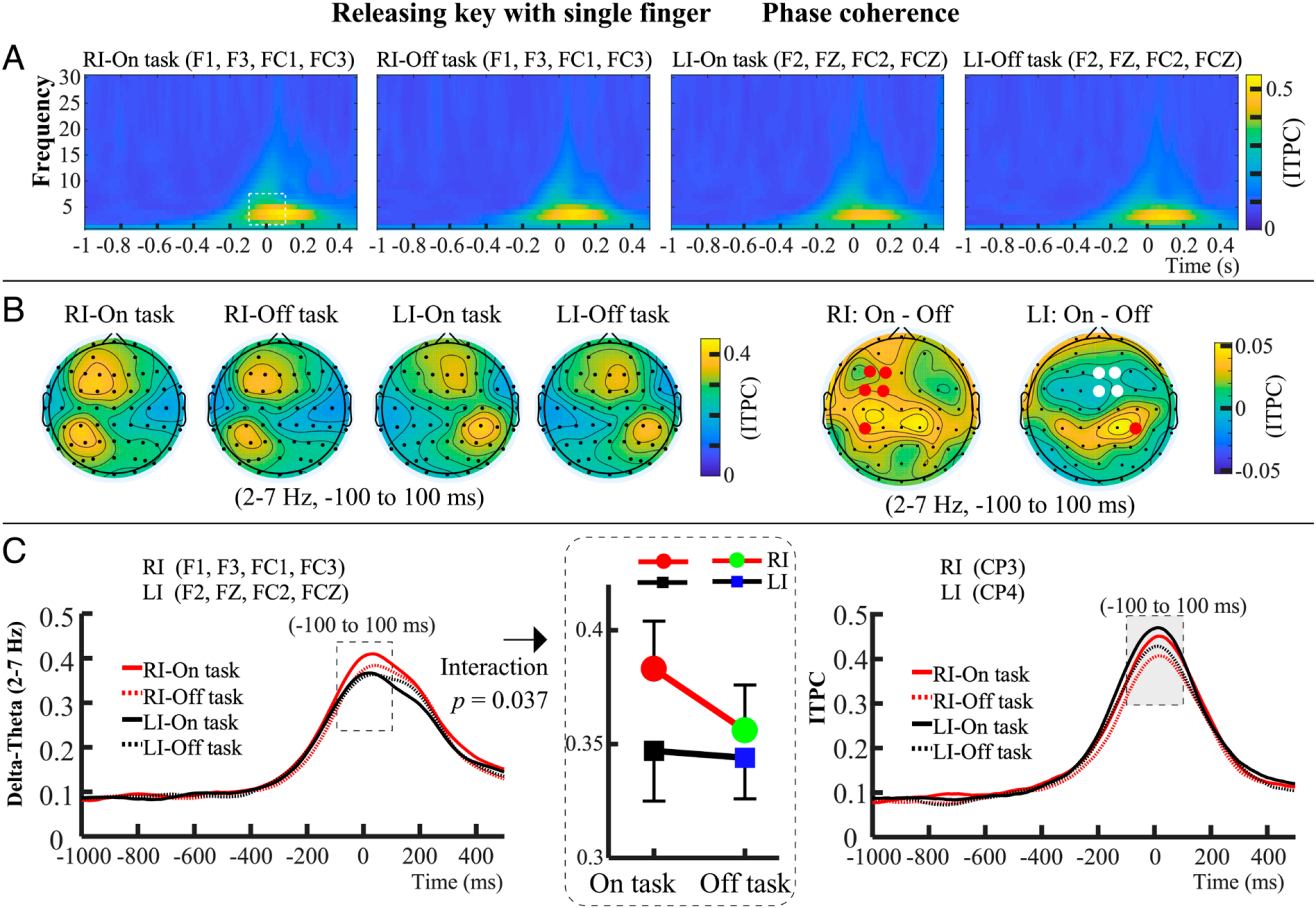
Phase coherence results of Experiment 3, the motor task of key-releasing with RI and LI. (*A*) Phase coherence of motor production occurred from 100 ms before to 100 ms after movement onset, primarily in the delta-theta band (2 to 7 Hz). The ROI was defined as this 200 ms time window, focusing on the delta-theta band, as shown by the white dashed box. (*B*) Phase coherence during RI key-releasing was observed at fronto-central electrodes (F1, F3, FC1, FC3) and centro-parietal CP3, while for LI key-releasing, it was observed at fronto-central electrodes (F2, FZ, FC2, FCZ) and centro-parietal CP4. ITPC data in the delta-theta band (2 to 7 Hz) from −100 to 100 ms were used to create topographical maps for each condition and their difference topographies. Electrodes used for statistical analysis are marked in red and white, with red indicating significant differences between “on task” and “off task” states. (*C*) For ITPC data from fronto-central electrodes (−100 to 100 ms; 2 to 7 Hz), mind wandering reduced phase coherence only in the RI condition, with no effect on the LI condition. For centro-parietal electrodes, mind wandering reduced phase coherence in both conditions.

Previously, Long et al. (10) extensively examined the relationship between 2 and 7 Hz activity and motor execution, highlighting that delta-theta activity associated with motor control is predominantly observed in the fronto-central and centro-parietal regions contralateral to the responding hand (10, 39–41). Based on this, we selected ITPC data from electrodes located in the contralateral fronto-central and centro-parietal regions for subsequent analysis (Fig. 4*B*). The time-frequency window of interest was defined as 2 to 7 Hz within 100 ms before and after the motor generation (Fig. 4*A*). The specific electrodes were selected by referencing the topographical map of the time-frequency window of interest, focusing on those exhibiting the highest ITPC values (Fig. 4*B*). For the ITPC in fronto-central region (F1, F3, FC1, FC3 electrodes for key-releasing with RI; F2, FZ, FC2, FCZ electrodes for key-releasing with LI), a 2 (probe: On task vs. Off task) × 2 (response hand: RI vs. LI) repeated-measures ANOVA on the average ITPC data (−100 to 100 ms, 2 to 7 Hz) revealed a marginal significant main effect of probe, *F*(1, 27) = 4.022, *P* = 0.055, *η_p_*^2^ = 0.130, a significant main effect of response hand, *F*(1, 27) = 4.835, *P* = 0.037, *η_p_*^2^ = 0.152, and a significant interaction between response hand and probe, *F*(1, 27) = 4.833, *P* = 0.037, *η_p_*^2^ = 0.152. Simple effect analysis showed that the delta-theta ITPC of RI-Off task was significantly lower than that of RI-On task (*P* = 0.034, *η_p_*^2^ = 0.268), whereas there was no significant difference between LI-On task and LI-Off task (*P* = 0.709, *η_p_*^2^ = 0.005). Noninferiority *t* tests with a margin of 0.01 were conducted. For the easier RI condition, the test was significant, *t*(27) = 2.14, *P* = 0.042, 95% CI [0.011, 0.045], indicating that mind wandering significantly reduced delta-theta ITPC. For the more difficult LI condition, the test was not significant, *t*(27) = −0.61, *P* = 0.544, 95% CI [−0.017, 0.025], indicating no significant effect of mind wandering on delta-theta ITPC. These results show that mind wandering only reduced the delta-theta phase coherence during motor generation for key-releasing with the easier RI, but not with the more difficult LI.

For the ITPC data (−100 to 100 ms, 2 to 7 Hz) in the centro-parietal region (CP3 electrode for key-releasing with RI, CP4 electrode for key-releasing with LI), there was a significant main effect of probe, *F*(1, 27) = 20.044, *P* < 0.001, *η_p_*^2^ = 0.426, but no significant main effect of response hand, *F*(1, 27) = 1.178, *P* = 0.287, *η_p_*^2^ = 0.042, and no significant interaction between response hand and probe, *F*(1, 27) = 0.005, *P* = 0.944, *η_p_*^2^ < 0.001. This result shows that mind wandering reduced delta-theta phase coherence during motor generation for both key-releasing tasks, the easier RI and the more difficult LI, at the centro-parietal region contralateral to the responding hand. In summary, in Experiment 3, the effect of mind wandering on phase coherence during motor generation was modulated by task difficulty, and this modulation was observed only in the contralateral fronto-central region.

### Experiment 4: Motor Processing in the More Difficult Motor Task of Key-Releasing with Right Index-Middle (RIM) and Right Index-Ring (RIR).

Experiment 4 introduced more challenging two-finger right-hand movements (RIM: index and middle fingers, RIR: index and ring fingers) to confirm that Experiment 3 findings were due to task difficulty. In a preliminary test (without thought probes), reaction times for both RIM and RIR were slower than RI, with no difference between RIM and RIR, indicating higher difficulty (Fig. 1*B* and *SI Appendix*, *Supplementary Results*). Off-task thoughts in RIM and RIR conditions were linked to slower reaction times and greater variability (Fig. 1*B*). Across all experiments, mind wandering consistently increased behavioral variability and, except in Experiment 1, also slowed reaction times. Mind wandering reports averaged ~50% across Experiment 1 to 4, with RIM (40.38%, *SE* = 1.16%) significantly lower than RIR (47.97%, *SE* = 1.54%) in Experiment 4 (*SI Appendix,* Fig. S2). This rate aligns with prior findings that sustained-attention tasks with 1-min probe intervals elicit ~50% mind wandering reports (43). Detailed results are in *SI Appendix*, *Supplementary Results*.

**Fig. 5.**
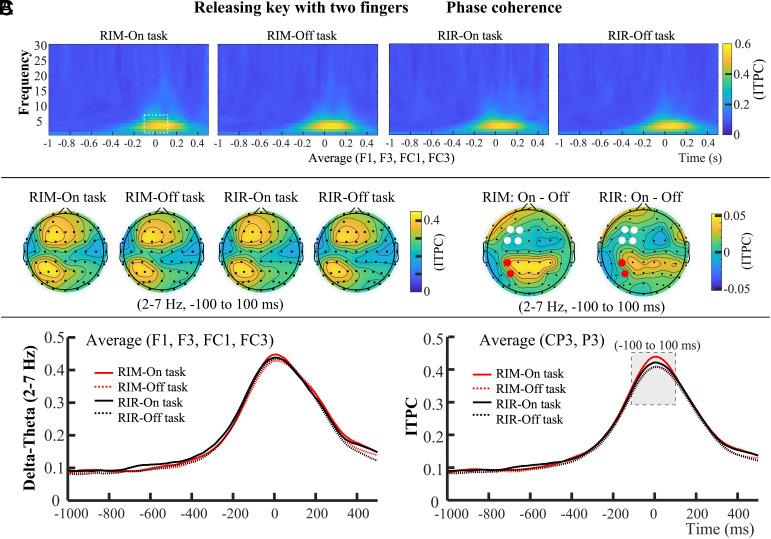
Phase coherence results of Experiment 4, the more difficult motor task of key-releasing with RIM and RIR. (*A*) Similar to Fig. 4, phase coherence related to motor generation occurred mainly in the delta-theta (2 to 7 Hz) band, spanning 100 ms before and after movement initiation. ITPC data in this frequency band and time window were used for statistical analyses, as shown by the white dashed box. (*B*) Phase coherence during RIM and RIR key-releasing was observed primarily at fronto-central electrodes (F1, F3, FC1, FC3) and centro-parietal electrodes (CP3, P3). In the difference topographies, electrodes used for statistical analysis are marked in red and white, with red indicating significant differences between “on task” and “off task” states. (*C*) For ITPC data from the fronto-central electrodes (−100 to 100 ms; 2 to 7 Hz), mind wandering did not affect phase coherence in either the RIM or RIR conditions. For centro-parietal electrodes, mind wandering reduced phase coherence in both conditions.

As in Experiment 3, we analyzed ITPC data from electrodes in the contralateral fronto-central and centro-parietal regions (Fig. 5*B*). The 2 to 7 Hz frequency range and the 100 ms time window before and after the movement execution were selected as the ROI (Fig. 5*A*), with specific electrodes chosen based on the topography of peak ITPC values (Fig. 5*B*). Both RIM and RIR in Experiment 4 were more difficult compared to RI in Experiment 3, that is, both RIM and RIR should be considered as relatively more difficult movements. We, therefore, expected that mind wandering would not affect phase coherence during the motor generation of these two movements. Since RIM and RIR were performed with the right-hand fingers, phase coherence of motor production (2 to 7 Hz) mainly occurred in the left hemispheric brain regions (Fig. 5*B*). For the ITPC data in fronto-central region (F1, F3, FC1, FC3 electrodes for key-releasing with RIM and with RIR), a 2 (probe: On task vs. Off task) × 2 (response type: RIM vs. RIR) repeated-measures ANOVA revealed no significant main effect of probe, *F*(1, 28) = 0.292, *P* = 0.593, *η_p_*^2^ = 0.01, no significant main effect of response type, *F*(1, 28) = 0.882, *P* = 0.356, *η_p_*^2^ = 0.031, and no significant interaction between response type and probe, *F*(1, 28) = 0.002, *P* = 0.967, *η_p_*^2^ < 0.001. This result suggests that mind wandering did not influence the delta-theta phase coherence of motor generation during the relatively more difficult movements (RIM and RIR) at the fronto-central region.

For the ITPC data in centro-parietal region (CP3, P3 electrodes for key-releasing with RIM and with RIR), there was a significant main effect of probe, *F*(1, 28) = 5.295, *P* = 0.029, *η_p_*^2^ = 0.159, but no significant main effect of response type, *F*(1, 28) = 0.500, *P* = 0.485, *η_p_*^2^ = 0.018, no significant interaction between response type and probe, *F*(1, 28) = 1.198, *P* = 0.283, *η_p_*^2^ = 0.041. This result indicates that mind wandering reduces the delta-theta phase coherence of motor generation even during relatively more difficult movements (RIM and RIR) at the centro-parietal region.

### The Negative Correlation between Phase Coherence and Behavioral Responses.

The ITPC serves as a neural proxy for temporal precision, measuring the degree of phase coherence in the brain’s neural activity during the poststimulus period, which can be influenced by external stimuli or tasks. How does this impact cognitive processing? Given its temporal nature, ITPC is expected to modulate the temporal features of cognitive processing, as reflected in reaction time: Shorter reaction times indicate more temporally precise cognitive processing. We thus anticipated a negative correlation between ITPC and behavioral reaction times: Higher ITPC indicates greater temporal precision on the neural level relative to the onset of the stimuli, and shorter reaction times also reflect higher temporal precision of cognitive processing. Additionally, given that ITPC is a variance-based measure, we hypothesized that it would also be linked to reaction time variability from trial to trial as calculated by its ICV (rather than the mean of reaction time as averaged across all trials). The variance of phase angles across trials may be related to the variance in reaction times. Therefore, we expected individuals with higher ITPC values to exhibit faster and/or less variable reaction times, reflecting a negative correlation between phase coherence and behavioral responses.

**Fig. 6.**
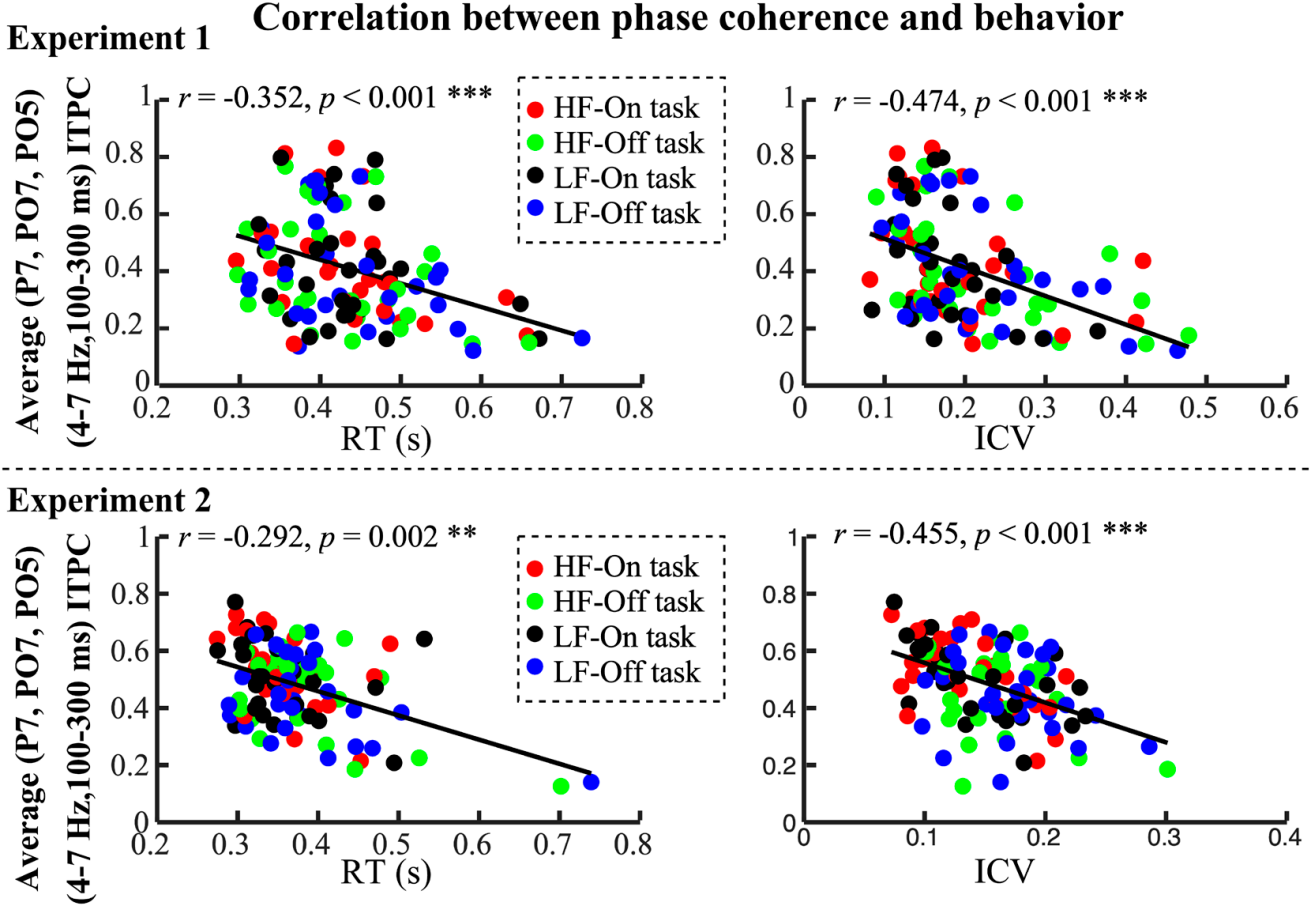
Correlation between phase coherence and behavioral responses in Experiments 1 and 2. During the sensory tasks in Experiments 1 and 2, the average ITPC in the 4 to 7 Hz range and 100 to 300 ms time window at the left occipital electrodes (P7, PO7, PO5) consistently showed negative correlations with both reaction times and reaction variability. Spearman’s correlation coefficient was used to assess these relationships.

To test this hypothesis, we computed the Spearman correlation coefficient between these variables. Across both Experiments 1 and 2, the average ITPC in the 4 to 7 Hz frequency range and 100 to 300 ms time window at the left occipital electrodes (P7, PO7, PO5) consistently showed a significant negative correlation with reaction time mean. In Experiment 1, the overall correlation was significant (*r* = −0.352, *P* < 0.001) (Fig. 6). Condition-specific analyses revealed significant correlations both in the HF-On (*r* = −0.477, *P* = 0.01) and HF-Off (*r* = −0.371, *P* = 0.048), while LF-On (*r* = −0.239, *P* = 0.212) and LF-Off (*r* = −0.352, *P* = 0.062) showed no significant relationships. Reaction variability (ICV of RT) also demonstrated a significant overall negative correlation with ITPC (*r* = −0.474, *P* < 0.001), with condition-specific correlations following a similar trend: HF-On (*r* = −0.520, *P* = 0.004), HF-Off (*r* = −0.562, *P* = 0.002), LF-On (*r* = −0.377, *P* = 0.044), and LF-Off (*r* = −0.427, *P* = 0.022).

In Experiment 2, a similar pattern emerged. The overall negative correlation between ITPC and reaction time mean was *r* = −0.292, *P* = 0.002 (Fig. 6). For condition-specific correlations, HF-On (r = −0.374, *P* = 0.046) showed significant correlation, while HF-Off (*r* = −0.229, *P* = 0.231), LF-On (*r* = −0.353, *P* = 0.061), and LF-Off (*r* = −0.276, *P* = 0.147) were not significant. Reaction variability (ICV of RT) also maintained a significant overall correlation (*r* = −0.455, *P* < 0.001), with strongest effects in HF-On (*r* = −0.511, *P* = 0.005) and LF-On (*r* = −0.684, *P* < 0.001), and weaker effects in HF-Off (*r* = −0.249, *P* = 0.192) and LF-Off (*r* = −0.229, *P* = 0.231). Additionally, analyses at the right occipital (PO6, PO8, P8) and central frontal (F1, FC1, FCZ) electrodes revealed consistent negative correlations of the ITPC with both reaction time mean and reaction time variability across conditions. Finally, a somewhat similar pattern emerged in our motor tasks. In Experiments 3 and 4, a significant negative correlation was observed between the average ITPC in the 2 to 7 Hz frequency range (within the −100 to 100 ms time window) and reaction time mean at the centro-parietal electrodes (*SI Appendix,* Fig. S6). Specifically, the overall correlation was *r* = −0.208, *P* = 0.028 in Experiment 3 and *r* = −0.314, *P* < 0.001 in Experiment 4, but condition-specific correlations were not significant. No negative correlation between ITPC and reaction variability (ICV of RT) was found in the motor tasks.

In summary, these findings indicate that higher ITPC values are linked to faster and more consistent/less variable reaction times; this further supports the idea that ITPC, as a measure of the temporal precision of phase variance at the neural level, indeed relates to temporal precision at the cognitive level, e.g., reaction time.

## Discussion

### Mind Wandering Modulates Only Easy Sensory and Motor Tasks but Not Difficult Ones.

This study aimed to investigate the impact of mind wandering on phase coherence during both sensory and motor tasks with varying degrees of task difficulty. Summarizing findings from all experiments, both in visual input and motor output processing, mind wandering reduced phase coherence (ITPC) only in easy tasks, without affecting difficult tasks. In Experiment 2, participants were instructed to prioritize task-relevant color information over task-irrelevant semantic information. We found that mind wandering reduced midfrontal theta (4 to 7 Hz) ITPC, which reflects the integration of choice-relevant information during goal-directed behavior (36–38), in the easy HF condition, but did not affect the more difficult LF condition. This finding indicates that even when processing task-irrelevant visual information, task difficulty still modulates the impact of mind wandering on phase coherence. In the motor tasks of Experiments 3 and 4, task difficulty also played a critical role in modulating the impact of mind wandering on the phase coherence of motor output processing. This effect was observed only in the fronto-central region contralateral to the responding hand. In contrast, in the contralateral centro-parietal region, mind wandering consistently reduced delta-theta phase coherence of motor generation, regardless of whether the movement was easy or difficult. This distinction may reflect functional differences between these two regions in terms of their relevance for motor generation (44). Here, we focus primarily on the contralateral fronto-central region, which is closely associated with motor control (10, 41, 44). Further discussion is provided in later sections.

### Phase Coherence as an Index of Temporal Precision Modulates the Effect of Mind Wandering on Sensory and Motor Processing.

Our study’s key finding that mind wandering reduces phase coherence in simple tasks may provide a neural mechanism explanation for the modulation of both easy sensory and motor processing by mind wandering. Input processing in the brain is regulated by phase synchronization (45). Incoming external stimuli reset the ongoing randomly distributed phase angles of neural oscillations, leading to the temporally precise clustering of phase angles within the hundreds-of-millisecond range following the onset of the stimulus when presented repeatedly and averaged over trials (46). Subsequently, ITPC serves as a measure for the temporal consistency in phase angles across trials at the time points of the sensory or motor stimuli, thus acting as a proxy indicator of both temporal precision and temporal segregation in relation to the temporal features of external inputs (25). In Experiments 1 and 2, higher ITPC values were associated with faster and more consistent (less variable) reaction times (temporal precision at the behavioral level), further supporting the idea that ITPC reflects the temporal precision of phase variance at the neural level. Given that phase coherence can be utilized to measure temporal precision (45), we speculate that attenuation of sensory processing during mind wandering may originate from temporal imprecision at the neuronal level, leading to the brain’s inability to temporally segregate single inputs from others through relating their distinct time points with distinct components of the ongoing phase cycles (problems with temporal segregation). This converges with the observation that mind wandering operates on longer timescales with longer and thus temporally less sharp or precise cycles at potentially slower frequencies (26, 47). Thus, due to its longer timescale (as related to slower frequencies with their longer phase cycles), mind wandering introduces temporal imprecision by disrupting the fine-grained synchronization necessary for processing external stimuli with high temporal resolution (21).

In addition to sensory input processing, motor output processing is also mediated by phase synchronization. Popovych et al. (41) found that delta-theta (2 to 7 Hz) phase locking most pronounced in M1 contralateral to the moving hand may ensure the distinct active cortical pathways to converge to the common motor output. Correspondingly, we found that mind wandering reduced delta-theta (2 to 7 Hz) ITPC during the execution of simple movements in our first motor task in Experiment 3 at the contralateral fronto-central electrodes. This may indicate that the brain fails to temporally segregate a single motor output from others with their distinct time points during mind wandering. Such a decrease in both temporal segregation and precision may lead the brain to be unable to accurately and effectively guide the activation of appropriate muscles to perform the corresponding actions. Hence, as aligned with Long et al. (10), mind wandering weakens the motor control over simple movements but does not influence the more complex control of difficult movements. These findings collectively extend the perceptual decoupling hypothesis (7, 11) from sensory to motor domains, suggesting that reduced phase coherence serves as a unifying neural mechanism underlying the decoupling from external processing across multiple cognitive domains. This temporal precision framework provides a mechanistic explanation for how mind wandering differentially affects information processing depending on task demands.

It is important to note that the modulatory effect of task difficulty on mind wandering’s influence on temporal precision was observed only at the neural level, not at the behavioral level. We speculate that this discrepancy arises because the neural temporal precision indicator, i.e., ITPC, is more temporally precise than behavioral indicators such as reaction time (RT) and its variability: The ITPC is sampled and thus measured in a high frequency range (EEG sampling rate: 1,000 Hz) whereas RT is sampled and measured in a much more coarse-grained way (Computer screen refresh rate: 120 Hz) and therefore remains more temporally imprecise due to its measurement method. This greater precision may allow ITPC to capture the effects of mind wandering more sensitively.

### Mind Wandering and Phase Coherence Only Modulate Easy but Not Difficult Sensory and Motor Tasks.

Why do only easy tasks, but not difficult ones, modulate the attenuation effect of mind wandering on phase coherence in visual input and motor output processing? According to the resource-control account of sustained attention (29), the presence of mind wandering is associated with a decrease in motivation or effort to sustain attention on the primary task as time progresses. Less complex tasks require less cognitive effort, which makes motivation more susceptible to decline and increases the likelihood that executive resources will be diverted toward mind wandering (1, 29, 48). Since rhythmic brain activity interacts in complex ways with both internal and external environments through “neuronal entrainment” (20), this diversion may lead to stronger phase coherence with internal cognition and reduced phase coherence with external stimuli. In contrast, necessitating greater allocation of available resources and thereby limiting the cognitive resources available for internal mind wandering (1). As a result, phase coherence with internal cognition weakens because resources are now redirected toward entrainment to external stimuli. In other words, highly demanding external tasks may more effectively constrain and limit internal processes related to mind wandering (1, 49). Conversely, easier tasks with fewer external demands are more likely to be strongly affected by internal mind wandering, which reduces the processing of external stimuli by diminishing their temporal precision, resulting in lower ITPC. Importantly, in Experiments 1 and 3, mind wandering reports did not differ significantly between easy and difficult task conditions under our experimental design (i.e., alternating blocks of simple and difficult tasks; see *SI Appendix,* Fig. S2). Since mind wandering frequency was comparable across conditions, the observed outcome differences likely stem from fluctuations in internal activity intensity or phenomenological content during mind wandering episodes.

Given that mind wandering affects easy and difficult cognitive tasks differently by modulating temporal precision (ITPC), one may speculate that the content of thoughts also differs between the two tasks. Easy tasks provide more cognitive resources for internal mind wandering, which may therefore become more complex, involving a variety of timescales and thought durations. In contrast, more difficult external tasks divert cognitive resources away from internal mind wandering, making these thoughts less complex, with more limited and potentially shorter timescales, such as shorter thought durations. Smallwood et al. (50) provided evidence for the impact of task load on the temporal structure of thoughts during mind wandering, showing that individuals engaged in less prospective mind wandering (thinking about the future) when the primary task was more demanding. To further investigate this hypothesis, future studies could examine whether mind wandering content becomes less cognitively complex under higher task demands. This could be assessed through task performance, thought sampling, the temporal duration and timescales of thoughts, and/or changes in brain activity associated with mind wandering.

### ITPC: Marker of Cognitive Resource Control.

Resource control may be closely related to the reciprocal balance of internal and external cognition (27) (dual vs. baseline model). If external cognition is more difficult, it will “pull away” the resources from internal cognition, like mind wandering, reducing the influence of the latter on the former. In the case of the low resource demanding easy tasks, internal cognition with mind wandering can draw more substantial resources and therefore more strongly impact the lower resource demanding easy tasks. Hence, in simpler tasks, where the demand for external cognitive processing is lower, individuals are more inclined to engage in internal task-unrelated thoughts, resulting in a decrease in the processing of external stimuli and subsequent enhancement of mind wandering (1, 48). However, as task difficulty increases and external cognitive processing demands rise, individuals may need to devote more attentional resources to the external task, shifting the balance of internal and external cognitive processing even in the presence of mind wandering. In this scenario, mind wandering and processing of external stimuli may progress concurrently in the background, with more attention devoted to the external stimuli in the foreground. In summary, mind wandering reduces phase coherence in simpler tasks, while having no effect on more difficult or challenging tasks—exactly what we observe in our data. ITPC may thus be considered a neural marker of the brain’s cognitive resource control across the demands of both internal (mind wandering) and external (tasks) cognition.

**Fig. 7.**
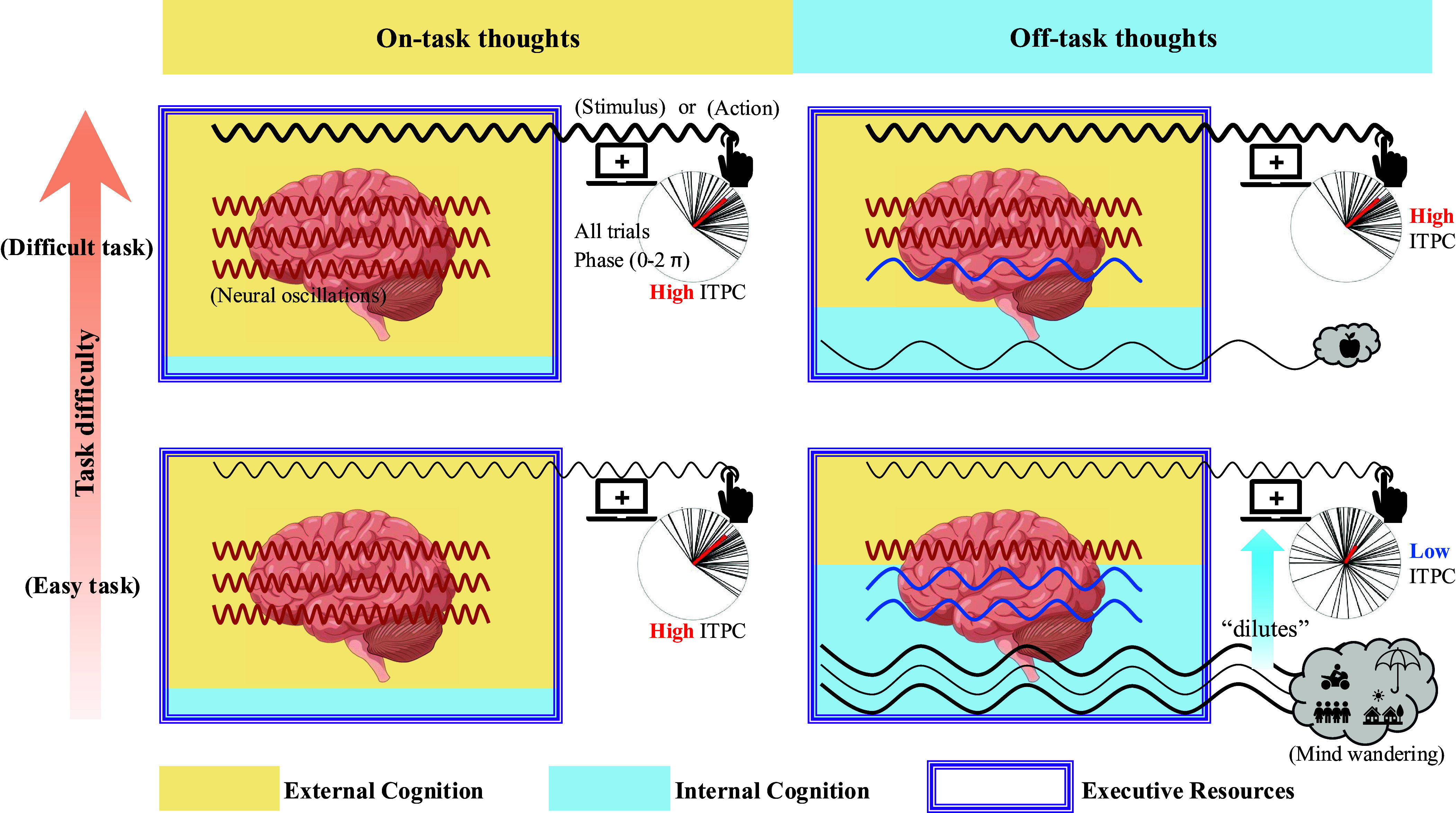
Schematic illustration of reciprocal balance between internal and external cognition and the modulating role of task difficulty on the effect of mind wandering on phase coherence. In demanding sensory or motor tasks, greater executive resource allocation to task execution (external cognition) reduces resources for mind wandering (internal cognition) (1, 28, 49, 53). Conversely, easier tasks require fewer executive resources, freeing more for mind wandering (1, 28, 49, 53). Mind wandering unfolds over longer timescales (26, 47), while externally driven stimulus processing occurs over shorter ones (21, 26). Thus, easier tasks facilitate mind wandering, which, due to its longer timescale (linked to slower frequencies and extended phase cycles), introduces temporal smoothing and imprecision at shorter timescales (associated with faster frequencies of external input). This reduces phase coherence between neural activity and external stimuli, leading to low entrainment and temporal imprecision, as reflected in low ITPC. In contrast, difficult tasks suppress mind wandering, preserving phase coherence and maintaining temporal precision (e.g., high ITPC). This dynamic highlights how executive resource allocation shapes temporal precision through phase angle coherence and entrainment, as measured by ITPC.

Together, it can be assumed that both internal and external cognition share common executive resources, with their internal-external balance modulating the strength of mind wandering and task difficulty. This is well in accordance with the recently proposed “Baseline model of internal and external cognition” where both are reciprocally interdependent upon each other rather than operating in parallel as in a Dual model (27). The Baseline model assumes a dynamic basis in the brain’s neural activity, which may account for its shared cognitive-executive resources for both internal and external cognition. This dynamic basis can be characterized at the neural level by phase coherence, which may be primarily driven by external cognition and exhibit high temporal precision in more difficult tasks. In contrast, during easy external tasks, the stronger impact of internal cognition with mind wandering may “dilute” the high phase coherence which then becomes temporally imprecise (Fig. 7). This phenomenon may, to some extent, manifest in individuals with schizophrenia, who exhibit high temporal imprecision in phase coherence during external tasks while simultaneously demonstrating heightened internal cognition, as seen in delusions, thought disorders, and hallucinations (25, 45, 51, 52).

### Methodological Limitations.

While our study observed task difficulty modulating the attenuation effects of mind wandering on phase coherence in both visual input and motor output processing, it is important to emphasize that the manipulation of task difficulty in our study was relative, that is, relative to the easy task, and we did not apply extremely difficult tasks where participants might fail to perform them. Therefore, we cannot determine the absolute threshold of task difficulty at which mind wandering ceases to affect phase coherence. Furthermore, we speculate that in exceedingly challenging tasks, mind wandering would also decrease phase coherence because individuals would be unable to attend to external events while mind wandering. Schizophrenia may be such a condition, as processing external information effectively is extremely difficult for these patients (25, 45, 51, 52, 54). A second limitation of our study is that our probes only required participants to provide binary reports: either on-task or off-task. As a result, we were unable to differentiate between various types of mind wandering, such as intentional and unintentional mind wandering (55). It is important to distinguish between intentional and unintentional mind wandering for several reasons, one of which is that the rates of these different types may vary with task difficulty (53, 55). Future research could further investigate whether this reduction in phase coherence during mind wandering in simple but not difficult tasks is due to its intentional nature, i.e., individuals may be more intentionally disengaging from the external environment.

## Conclusion

We investigated the effects of task difficulty in various external sensory and motor tasks on phase coherence and internal cognition, e.g., mind wandering. We observed that mind wandering exerted stronger effects on electrophysiological indices in simple sensory and motor tasks compared to more difficult tasks, where the same neural measures remained largely unaffected. Moreover, we observed that this effect was mediated by phase coherence, e.g., ITPC, relative to the external stimuli, which decreased only in easy tasks, but not in difficult ones. Importantly, these effects were observed in both sensory and motor tasks, thus suggesting an underlying shared cross-modal neural basis for both sensory and motor processing in the gestalt of phase dynamics as measured by ITPC. More broadly, our results suggest a reciprocal balance and dependence between internal cognition, e.g., mind wandering, and external cognition, e.g., sensory and motor tasks. This reciprocal balance of internal and external cognition seems to be, in part, mediated by phase dynamics, that is, specifically its temporal precision relative to the timing of the external stimuli. Phase dynamics may thus be a commonly shared neural substrate for both internal and external cognition, as proposed by the Baseline model of cognition (27). Given the well-known disruption of phase dynamics in schizophrenia (54), as well as the extreme shift toward internal cognition in depression (56–59), our behavioral and neural findings carry important implications beyond the healthy brain for understanding the neurodynamic basis of abnormal cognition in mental disorders.

## Methods

The procedures used for data acquisition and analysis are described in detail in *SI Appendix*, *Supplementary Methods*. A summary of the key steps is provided below.

### Experimental Design and Task.

Experiments 1 to 4 aimed to investigate how task difficulty influences mind wandering and its impact on phase coherence in both sensory and motor tasks. Experiment 1 involved a semantic classification task, where participants judged whether words were animal-related, pressing a key for nonanimal words and refraining for animal words, following the SART paradigm. HF and LF words induced varying levels of task difficulty. In Experiment 2, the task complexity was reduced to color classification, where participants focused on the color of words instead of their meaning, thereby making the task easier compared to Experiment 1. Experiment 3 shifted to a motor task, comparing simple (RI finger) and more challenging (LI finger) key-releasing movements. Handedness was used to manipulate the difficulty, with RI being easier and LI more difficult. Finally, Experiment 4 increased movement difficulty by introducing two-finger key-releasing tasks (RIR and RIM), which required more complex motor actions. All experiments included thought probes to assess mind wandering and used a block design with alternating task conditions.

### EEG and Behavioral Data Analysis.

#### EEG data acquisition and preprocessing.

EEG data were collected using 64 electrodes and preprocessed with EEGLAB (60), including rereferencing, bandpass filtering, and epoch extraction (1,500 ms for Experiments 1 and 2, 2,200 ms for Experiments 3 and 4). Artifact trials removal was performed manually, and eye blink/movement artifacts were corrected using a two-step ICA procedure (61).

#### Trials included in the analysis.

In all experiments, the six trials before thought probes were classified as on-task or off-task based on participants’ reports. Data were grouped accordingly for each experiment (e.g., HF-On task, HF-Off task, LF-On task, LF-Off task for Experiments 1 and 2; RI-On task, RI-Off task, LI-On task, LI-Off task for Experiment 3, etc.). To assess mind wandering, we analyzed behavioral response variability using the ICV, which measures the SD of reaction time divided by the mean reaction time.

#### ITPC analysis.

ITPC analysis (23, 62) was applied to determine the phase coherence of brain activity across different experimental conditions.

#### Relation between ITPC and behavioral responses.

Spearman correlation was used to evaluate the relationship between ITPC and both reaction time and its variability.

#### Significance testing.

All significance testing was performed using a two-factor repeated-measures ANOVA. The ITPC data for the analysis were selected based on prior knowledge, focusing on specific electrodes, frequency bands, and time windows. If a significant interaction effect was found, a simple effects analysis and a noninferiority *t* test were conducted to compare the differences between On-task and Off-task states across the two conditions, assessing practical significance.

## Supplementary Material

Appendix 01 (PDF)

## Data Availability

Anonymized EEG and behavioral data have been deposited in the online repository ITPC and mind wandering: Task difficulty modulates the effect of mind wandering on phase dynamics (https://osf.io/pj527/) (63).
