# Supplementary material for: Task difficulty modulates the effect of mind wandering on phase dynamics
Source: Proc Natl Acad Sci U S A. 2025 May 30;122(22):e2416387122. doi: 10.1073/pnas.2416387122 (PMC12146758; doi:10.1073/pnas.2416387122)
Supplement: Supplementary file 1 — Appendix 01 (PDF) [file pnas.2416387122.sapp.pdf]

Supporting Information for
Task difficulty modulates the effect of mind wandering on phase dynamics.

Zhengkun Long [1,2,3*], Georg Northoff [4#], Xiaolan Fu [1,2,5#*]

[1]State Key Laboratory of Cognitive Science and Mental Health, Institute of Psychology, Chinese Academy of Sciences, Beijing, 100101, China

[2]Department of Psychology, University of Chinese Academy of Sciences, Beijing, 100049, China

[3]School of Psychology, Shenzhen University, Shenzhen, Guangdong, China

[4]Mind, Brain Imaging and Neuroethics Research Unit, The Royal's Institute of Mental Health Research, University of Ottawa, Ottawa, ON, Canada

[5]School of Psychology, Shanghai Jiao Tong University, Shanghai, 200030, China

[#] Contributed equally.

* Corresponding author: Zhengkun Long, Xiaolan Fu.

Email: longzk@psych.ac.cn, fuxiaolan@sjtu.edu.cn

This PDF file includes:

Supplementary Methods
Supplementary Results
Figures S1 to S6
SI References

Supplementary Methods

Participants. G*Power 3.1.9.7 software (1) was used to estimate the sample size. As a two-way repeated-measures ANOVA was used in all experiments of the current study, an alpha level of $\alpha = 0.05$, an effect size of 0.25, and $\rho = 0.5$ were adopted. For each of the four experiments, the total sample size required to achieve a statistical power level of 0.8 was determined to be 24. The number of participants in the four experiments of this study was 32, 29, 30, and 29, respectively. They had normal or corrected-to-normal vision and reported no history of neurological illness or head injury resulting in loss of consciousness. All participants were right-handed and provided written informed consent. Each participant received 120 RMB for their participation. The study received approval from the Ethics Committee of the Institute of Psychology, Chinese Academy of Sciences.

Experimental design and task (Experiment 1, semantic classification task). Experiment 1 aimed to establish two conditions in a semantic classification task: one simple and one challenging, to investigate how task difficulty affects the impact of mind wandering on phase coherence in visual input processing. EEG data from Long et al. (2) were used, where high- and low-frequency words induced varying levels of task difficulty in a sustained attention to response task (SART). Thirty-two university students (21 women; M_{age} = 21.94 years, SD_{age} = 2.22 years) participated, with data from three excluded due to poor performance (more than 3 SDs below the mean accuracy). The task involved 805 high- and 805 low-frequency non-animal words as nontargets and 45 high- and 45 low-frequency animal words as targets. A block design alternated high- and low-frequency word blocks, each ending with a probe. The experiment included 840 nontarget trials, 45 target trials, and 45 probes for high- and low-frequency blocks separately, totaling 1860 trials. This experimental design, which alternates blocks of varying difficulty levels, requires participants to perform tasks of different demands in succession, thereby minimizing the potential discrepancy in mind-wandering report ratios between simple and challenging tasks (3, 4).

Participants viewed a series of Chinese words presented sequentially on a gray screen. Each word appeared for 500 ms, followed by a 1300–1700 ms fixation cross. Words subtended 1 degree of visual angle. Participants pressed the “K” button with their left index finger as quickly and accurately as possible for nontarget words (non-animal) and refrained from pressing for target words (animal). Probes, asking about attention focus, appeared randomly. Participants chose between “on task” or “off task.” Targets and probes were presented pseudo randomly, with at least six consecutive nontarget trials between them. Probes appeared every 50 seconds, with intervals ranging from 12 to 80 seconds. This design followed Seli et al. (5), who found that about 50% of mind wandering was reported when probes were presented at a rate of one per minute. The experiment consisted of six sessions, each lasting about 13 minutes, with a minimum 2-minute rest between sessions. Before starting, participants completed 120 practice trials with instructions and feedback to ensure task understanding. They were informed that off-task reports referred to attention shifting from the task to unrelated thoughts or feelings, considered endogenous and arising from memory, imagination, or plans. Participants were also told they didn’t need to stop their thoughts immediately upon noticing task-unrelated ones. Further details on the experiment design and its facilitation of mind wandering are provided in Long et al. (2).

Experimental design and task (Experiment 2, color classification task). Experiment 2 aimed to build on Experiment 1 by reducing task difficulty to explore how mind wandering affects phase coherence in visual input processing of easier tasks. In this experiment, nontarget words were displayed in blue, red, green, or black, while target words were in white, and participants focused on the color rather than word meaning. Twenty-nine university students (17 women; M_{age} = 22.45 years, SD_{age} = 2.37 years) participated. They pressed the “K” button when words appeared in color and refrained when they were white. All other aspects of the design were consistent with Experiment 1, with both high- and low-frequency words conditions categorized as easy tasks.

Experimental design and task (Experiment 3, motor task of key-releasing with RI and LI). Experiment 3 aimed to examine the impact of movement difficulty on the effect of mind wandering on phase coherence in motor generation. Two conditions were created using handedness to manipulate movement difficulty (6), with simpler movements involving the right index finger (RI) and more challenging movements using the left index finger (LI). Thirty university students (19

women; M_{age} = 24.07 years, SD_{age} = 2.42 years) participated. Data from two participants were excluded: one due to technical issues with the program and one due to having fewer than 40 trials in one condition. A block design was used, alternating between 45 RI and 45 LI blocks ("RLLRRL"), with a probe at the end of each block asking whether participants were "on task" or "off task." Each block contained 6 to 21 trials, with an average probe interval of 60 seconds (ranging from 18 to 84 seconds). The experiment included 1040 trials, with 520 trials in both the RI and LI blocks. Each trial began with a 1500–2000 ms empty screen, followed by a white fixation cross. Participants pressed the "P" key (for RI) or "Q" key (for LI) with the right or left index finger as quickly as possible, holding the key until the fixation cross changed color (1500–2000 ms). Upon the color change, participants released the key as quickly as possible. The fixation cross (red, blue, or green) disappeared immediately after key-release, initiating the subsequent trial. The experiment consisted of six sessions, each with 15 blocks, separated by at least a two-minute rest break. Prior to the experiment, participants completed 80 practice trials. Further experimental details are provided in Long et al. (6). Key-releasing movements were the primary focus, with RI corresponding to the easy task and LI to the difficult task.
Experimental design and task (Experiment 4, more difficult motor task of key-releasing with RIR and RIM). Since RI key-releasing in Experiment 3 was considered a simple movement (6), Experiment 4 aimed to build upon this by introducing two-finger movements to increase the movement difficulty. This was done to investigate how mind wandering affects the phase coherence of motor generation in more challenging motor tasks. Experiment 4 was also part of the recent study conducted by Long et al. (6). Twenty-nine university students (18 women; M_{age} = 22.79 years, SD_{age} = 2.20 years) participated voluntarily. The experimental design mirrored Experiment 3, with the only difference being the fingers used. In RIM blocks, participants simultaneously pressed the "I" and "O" keys with the right index and middle fingers; in RIR blocks, they simultaneously pressed the "I" and "P" keys with the right index and ring fingers.

EEG Data Acquisition and Preprocessing. EEG data were collected using 64 Ag/AgCl scalp electrodes, which were placed according to the International 10-20 system (Neuroscan Inc., Charlotte, North Carolina, USA). The left mastoid served as the online reference. All electrodes maintained an impedance lower than 5 kΩ. Signals were sampled at 1000 Hz with a 0.01–100 Hz bandpass filter (SynAmps 4.5, Neuroscan, Inc., Charlotte, NC, USA). EEG data were processed using EEGLAB (7), an open-source MATLAB-based toolbox. The continuous EEG data were first re-referenced with the average reference technique. The continuous EEG data were then bandpassed at 0.1–30 Hz using a conventional finite impulse response (FIR) filter. For Experiments 1 and 2, EEG epochs of 1500 ms (500 ms before and 1000 ms after word onset) were extracted, with baseline correction from the pre-stimulus interval (-200 to 0 ms). For Experiments 3 and 4, epochs of 2200 ms (1700 ms before and 500 ms after key release) were used, with baseline correction from the pre-movement interval (-1500 to -1100 ms). The EEG trials with artifacts were discarded manually, with the average proportion discarded as follows: Experiment 1, M = 3.28%, SE = 0.72%; Experiment 2, M = 1.58%, SE = 0.50%; Experiment 3, M = 0.84%, SE = 0.39%; Experiment 4, M = 0.33%, SE = 0.09%. To correct for eye blink and movement artifacts, we applied a two-step ICA procedure. First, data were high-pass filtered at 1 Hz to optimize ICA-based artifact rejection, in line with recommendations from Rodrigues et al. (8). ICA was conducted using the extended Infomax algorithm. Next, the ICA weights and spatial filters obtained from the 1 Hz filtered data were transferred back to the 0.1 Hz filtered data, where artifact components were identified and removed manually. The average number of independent components (ICs) removed in each experiment is as follows: Experiment 1, M = 7.79, SE = 0.79; Experiment 2, M = 7.62, SE = 0.55; Experiment 3, M = 8.54, SE = 0.45; Experiment 4, M = 7.17, SE = 0.46. This procedure effectively removed artifacts while preserving low-frequency information.

Data Analyses. In all experiments, the six trials before thought probes were classified as on-task or off-task based on participants' reports, following our previous methodology (2, 6). In Experiments 1 and 2, the six nontarget trials before probes were either low-frequency (LF) or high-frequency (HF) words, resulting in four data groups: HF-On task, HF-Off task, LF-On task, and LF-Off task. Although the same abbreviations were used for different conditions in both

experiments, the tasks varied. The average trial numbers for each group in Experiment 1 were reported in Long et al. (2). For Experiment 2, the mean number of EEG epochs for each group were: HF-On task, *M* = 139.41, *SD* = 26.69; HF-Off task, *M* = 121.48, *SD* = 27.85; LF-On task, *M* = 143.31, *SD* = 33.35; and LF-Off task, *M* = 117.55, *SD* = 30.38. In Experiment 3, the six trials before probes were grouped by subjective reports and response hands: RI-On task, RI-Off task, LI-On task, and LI-Off task. In Experiment 4, trials were grouped as RIM-On task, RIM-Off task, RIR-On task, and RIR-Off task. Average trial numbers for Experiments 3 and 4 were reported in Long et al. (6). To test whether "off-task" reports reflected mind wandering, we examined whether behavioral response variability increased, measured by the intraindividual coefficient of variation (ICV), the standard deviation of reaction time divided by the mean reaction time (2, 6).

Inter-trial Phase Coherence (ITPC) Analysis. Due to the impact of trial quantity on ITPC computation, after EEG data preprocessing, the number of trials for all participants across all experimental conditions was ensured to exceed 40. To ensure that the ITPC calculations were not influenced by unequal trial numbers across conditions, we balanced the number of trials for each participant across all conditions. Specifically, we randomly subsampled trials to match the condition with the lowest trial count for each participant. This approach allowed for within-subject comparisons without introducing biases related to unequal trial numbers, ensuring the robustness of our ITPC analyses. The phase angles of the oscillatory activity at each time point and frequency bin were extracted from the preprocessed EEG data. This was achieved by calculating the instantaneous phase of the signal using complex Morlet wavelets (9). The number of cycles for the Morlet wavelet was 4, balancing frequency and temporal resolution. Complex Morlet wavelets were computed for the full epoch length (-500 to 1000 ms for Experiment 1 and 2, -1700 to 500 ms for Experiment 3 and 4) between 1 and 30 Hz. Subsequently, the phase angles across trials within each data group were aggregated to compute the ITPC values (9, 10) according to equation (1):

$$ITPC_{tf} = \left| \frac{1}{N} \sum_{n=1}^{N} e^{ik_{tfn}} \right| \quad (1)$$

where *N* is the number of trials, *n* is the trial number, e^{ik} is Euler's formula providing the complex polar representation of a phase angle *k* on trial *n* at time-frequency point *tf*. The absolute value bars indicate the length of the average vector. This involved averaging the phase angles across trials at each time point and frequency bin. To quantify the phase consistency across trials, the circular statistics method was employed to calculate the length of the mean resultant vector, which represents the degree of phase alignment or coherence across trials. Higher ITPC values indicate greater phase coherence or synchronization of neural oscillations across trials, whereas lower ITPC values suggest more variable phase relationships.

Statistical Analysis. To select ITPC data for ANOVA analysis, we followed a general principle of electrode selection based on prior knowledge. We focused on the frequency band with the highest ITPC, particularly those with band-specific effects in sensory and motor domains. The time-frequency window was determined from time-frequency plots. In Experiment 1, participants judged semantic information, so we selected ITPC data from left occipital electrodes (P7, PO7, PO5), known to be related to word recognition (11, 12). We analyzed the 4–7 Hz theta band and the 100–300 ms time window, based on prior research linking theta with language and memory processes (13). A 2 (probe: On task vs. Off task) × 2 (word frequency: HF vs. LF) repeated-measures ANOVA was conducted using average ITPC values within the ROI. If the interaction effect was significant, we conducted a simple effects analysis and a non-inferiority t-test to compare differences between On-task and Off-task states for HF and LF conditions, assessing practical significance. Experiment 2 followed the same design as Experiment 1, but participants judged color instead of semantic information. ITPC data were analyzed from occipital electrodes (left: P7, PO7, PO5; right: P8, PO8, PO6), linked to visual processing (14). As in Experiment 1, the strongest phase coherence in visual input processing occurred in the theta band (4–7 Hz) within 100–300 ms after word presentation. Since color judgment is simple but may be influenced by automatic semantic activation, we also analyzed midfrontal theta ITPC (4–7 Hz), associated with integrating choice-relevant information during goal-directed behavior (15–17). Using F1, FC1, and FCZ electrodes, we performed a two-factor repeated-measures ANOVA on theta ITPC data within 100–300 ms.

Experiments 3 and 4 focused on motor tasks. Popovych et al. (18) found phase locking in the delta-theta (2–7 Hz) band at the contralateral motor cortex is crucial for motor production, and Long et al. (6) observed similar activity in contralateral fronto-central and centro-parietal regions. Based on these findings, we selected ITPC data from electrodes in these regions, focusing on the 2–7 Hz band within 100 ms before and after motor generation. In Experiment 3, we analyzed ITPC data from fronto-central F2, FZ, FC2, FCZ, and centro-parietal CP4 for LI key-releasing, and from F1, F3, FC1, FC3, and centro-parietal CP3 for RI key-releasing using a 2 (probe: On vs. Off task) × 2 (response hand: RI vs. LI) repeated-measures ANOVA. In Experiment 4, since all key-releasing movements were with the right hand, ITPC data from fronto-central F1, F3, FC1, FC3 electrodes, and centro-parietal CP3 and P3 electrodes were selected for analysis.
As ITPC was considered a measure of temporal precision with respect to the timing and onset of the external stimuli, we hypothesized that it would be associated with behavioral reaction times and variability as both indicate temporal precision (shorter RT, lower RT variability) on the behavioral level. Since ITPC reflects the variance in phase angles of signals across trials, we expected individuals with higher ITPC values to exhibit faster and less variable reaction times. To test this, we calculated the Spearman correlation coefficient between ITPC and both reaction time and its variability.

Supplementary Results

Behavioral results of Experiment 1. The aim of this study was to explore the effects of mind wandering on phase coherence in sensory and motor tasks of varying difficulty. To achieve this research goal, we needed to design easy and difficult task conditions in the experiment. In Experiment 1, we manipulated task difficulty using word frequency. Participants were required to judge whether the words appearing on the screen belonged to animal words. Evidence that the task difficulty of low-frequency words was significantly higher than that of high-frequency words came from their slower reaction times and the elicitation of larger N400 components. We also rated the familiarity of all the words before the experiment. The familiarity ratings were significantly lower for the low-frequency non-target words (M = 4.12, SD = 0.96) than for the high-frequency non-target words (M = 5.78, SD = 0.67), Wilcoxon's signed rank test: $z = -23.33$, $p < 0.001$. For reaction times in Experiment 1, a 2 (Word Frequency: high vs. low) × 2 (Probe: on task vs. off task) repeated-measures ANOVA revealed a significant main effect of Word Frequency, $F(1, 28) = 6.57$, $p = 0.016$, $\eta_p^2 = 0.190$, but no significant main effect of Probe, $F(1, 28) = 0.18$, $p = 0.68$, $\eta_p^2 = 0.006$, and no significant interaction between Word Frequency and Probe, $F(1, 28) = 1.72$, $p = 0.20$, $\eta_p^2 = 0.058$. For N400 amplitudes (averaged across C1, CZ, C2, CP1, CPZ, CP2, P1, PZ, P2 electrodes; 300–500 ms) in Experiment 1, a 2 (Word Frequency: high vs. low) × 2 (Probe: on task vs. off task) repeated-measures ANOVA revealed a significant main effect of Word Frequency, $F(1, 28) = 17.19$, $p < 0.001$, $\eta_p^2 = 0.38$, a significant main effect of Probe, $F(1, 28) = 16.42$, $p < 0.001$, $\eta_p^2 = 0.37$, and a significant interaction between Word Frequency and Probe, $F(1, 28) = 4.84$, $p = 0.036$, $\eta_p^2 = 0.147$. Together, these results indicate that manipulating task difficulty using word frequency is effective, with low-frequency words representing difficult task conditions and high-frequency words representing easy task conditions. In Experiment 1, mind wandering ("off task") led to more variable behavioral responses, a 2 (Word Frequency: high vs. low) × 2 (Probe: on task vs. off task) repeated-measures ANOVA revealed a significant main effect of Probe, $F(1, 28) = 12.81$, $p = 0.001$, $\eta_p^2 = 0.314$, but no significant main effect of Word Frequency, $F(1, 28) = 0.033$, $p = 0.86$, $\eta_p^2 = 0.001$, and no significant interaction between Word Frequency and Probe, $F(1, 28) = 0.001$, $p = 0.97$, $\eta_p^2 = 0.001$. For more detailed statistical analyses of Experiment 1, please refer to Long et al. (2).

Behavioral results of Experiment 2. In Experiment 2, we used the same experimental design as in Experiment 1, except that we asked participants to judge the color in which the words were presented rather than focusing on their semantics. To assess how mind wandering influences reaction times, a 2 (probe: On task vs. Off task) × 2 (word frequency: HF vs. LF) repeated-measures ANOVA was employed on RT data. Reaction times in Experiment 2 were generally faster than those in Experiment 1, indicating that the overall color judgment task in Experiment 2 was easier. Thus, both conditions in Experiment 2 ("HF" and "LF") should be considered relatively easy tasks compared to especially the more difficult task (LF) in Experiment 1. Concerning the reaction times in Experiment 2, we observed a significant main effect of probe (on vs. off), $F(1, 28) = 13.437$, $p = 0.001$, $\eta_p^2 = 0.324$. While no significant main effect of word frequency (high vs. low frequency), $F(1, 28) = 0.020$, $p = 0.888$, $\eta_p^2 < 0.001$, nor a significant interaction between probe and word frequency, $F(1, 28) = 0.275$, $p = 0.604$, $\eta_p^2 = 0.010$, was observed. Accordingly, the findings show that mind wandering led to slower reaction times during off-task thoughts compared to on-task thoughts, suggesting that mind wandering modulates reaction time in this simple sensory task. While there was no significant difference in reaction times between the high- and low-frequency word conditions, this mirrors the smaller, if not absent, difference between the HF and LF conditions in the color classification task of Experiment 2, compared to the difference observed in Experiment 1. To assess whether mind wandering caused more variable behavioral responses, a 2 (probe: On task vs. Off task) × 2 (word frequency: HF vs. LF) repeated-measures ANOVA was employed on ICV of RT data. It revealed a significant main effect of probe, $F(1, 28) = 22.393$, $p < 0.001$, $\eta_p^2 = 0.444$, and a significant main effect of word frequency, $F(1, 28) = 5.763$, $p = 0.023$, $\eta_p^2 = 0.171$, but no significant interaction between probe and word frequency, $F(1, 28) = 1.111$, $p = 0.301$, $\eta_p^2 = 0.038$. The increase in reaction time variability suggests that mind wandering as indexed by "off task" reports in Experiment 2 modulates reaction time in a more variable way than on-task. Further, we found that the reaction variability for low-frequency words was significantly greater than for high-frequency words, possibly indicating stronger semantic interference from low-frequency words. This suggests that, although semantic information was task-irrelevant, the difference in semantic processing difficulty between low-frequency and high-frequency words may still have an impact.

Judging color is a relatively simple task; thus, both low-frequency and high-frequency word conditions in Experiment 2 should fall under a simple task environment. However, since word meaning is automatically activated, semantics in Experiment 2 were task-irrelevant information that might interfere with task performance. The interference effect of the task-irrelevant low-frequency word semantics on the task should be stronger than that of high-frequency words. In summary, Experiment 1 can examine the effects of mind wandering on phase coherence in easy and difficult sensory tasks. In addition to examining the effects of mind wandering on phase coherence in simple sensory tasks, Experiment 2 can also investigate the impact of mind wandering on the interference effect of task-irrelevant information of varying difficulty.

Behavioral results of Experiment 3. In Experiment 3, we manipulated movement difficulty using handedness. We required right-handed participants to perform key-releasing movements using their right index finger (RI) and left index finger (LI). The validity of the movement difficulty manipulation was verified using reaction time. The speed of key-releasing with LI was significantly slower than with RI, a 2 (probe: on task vs. off task) × 2 (response hand: RI vs. LI) repeated-measures ANOVA revealed that there was a significant main effect of probe, $F(1, 27) = 21.552$, $p < 0.001$, $\eta_p^2 = 0.444$, and a significant main effect of response hand, $F(1, 27) = 7.290$, $p = 0.012$, $\eta_p^2 = 0.213$, but no significant interaction between probe and response hand, $F(1, 27) = 0.093$, $p = 0.763$, $\eta_p^2 = 0.003$. These results indicate that key-releasing with LI was significantly more difficult than with RI. In Experiment 3, mind wandering ("off task") led to more variable behavioral responses, a 2 (probe: on task vs. off task) × 2 (response hand: RI vs. LI) repeated-measures ANOVA on ICV data revealed that there was a significant main effect of probe, $F(1, 27) = 9.571$, $p = 0.005$, $\eta_p^2 = 0.262$, and a significant main effect of response hand, $F(1, 27) = 5.587$, $p = 0.026$, $\eta_p^2 = 0.171$, but no significant interaction between probe and response hand, $F(1, 27) = 0.023$, $p = 0.881$, $\eta_p^2 < 0.001$.

Behavioral results of Experiment 4. However, considering the potential differences in motor control between left- and right-hand actions, Experiment 4 increased movement difficulty based on key-releasing with RI from Experiment 3 by designing dual-finger movements, which should be more difficult than single-finger movements. Experiment 4 required participants to perform key releases simultaneously with the right index and middle fingers (RIM), as well as with the right index and ring fingers (RIR). Before the formal experiment, we asked 21 participants (11 women; M = 22.38 years, SD = 2.03 years) to test the reaction speeds of RI, RIM, and RIR key releases in a behavioral experiment without thought probe. One-way repeated-measures ANOVA for RI, RIM, and RIR revealed a significant effect of condition on reaction time, $F(1.36, 27.22) = 10.207$, $p = 0.002$, $\eta_p^2 = 0.338$. Further pairwise comparisons showed that reaction time for RIM (M = 0.322 s, SE = 0.008) was significantly slower than for RI (M = 0.308, SE = 0.009), $p = 0.019$, and for RIR (M = 0.330, SE = 0.009) was also slower than for RI, $p < 0.001$, but there was no significant difference between RIM and RIR, $p = 0.379$. For reaction times in Experiment 4, a 2 (probe: on task vs. off task) × 2 (response type: RIM vs. RIR) repeated-measures ANOVA revealed that only the main effect of probe reached significance, $F(1, 28) = 50.140$, $p < 0.001$, $\eta_p^2 = 0.642$, there was no significant main effect of response type. RIM key-releasing and RIR key-releasing showed no difference in movement difficulty, and they were more difficult than single-finger movements (RI) in Experiment 3. Therefore, both RIM and RIR key releases should be considered relatively difficult movements. In Experiment 4, mind wandering ("off task") led to more variable behavioral responses, a 2 (probe: on task vs. off task) × 2 (response type: RIM vs. RIR) repeated-measures ANOVA on ICV data revealed that there was only a significant main effect of probe, $F(1, 28) = 30.190$, $p < 0.001$, $\eta_p^2 = 0.519$. In summary, Experiment 3 can examine the effects of mind wandering on phase coherence in easy and difficult motor tasks. Experiment 4 can further investigate the effects of mind wandering on phase coherence in difficult motor tasks. For more detailed statistical analyses of Experiment 3 and 4, please refer to Long et al. (6).

Off-task reports provided the most direct reflection of mind wandering. In all four experiments of this study, we found that off-task thoughts were associated with more variable behavioral responses, which supports behavioral variability as a stable indicator of mind wandering. However, the effect of mind wandering on reaction times was not consistent across experiments. In Experiment 1, we did not observe an effect of off-task thoughts on reaction times, but in the other three experiments, mind wandering led to slower reaction times. We attribute this difference to the task type. The key distinction between Experiment 1 and the others lies in the more complex

cognitive processing required before making a motor response (e.g., semantic processing in Experiment 1). The overall reaction times in Experiment 1 were the longest among the four experiments, suggesting that the complexity of cognitive processing before responding plays a role. In contrast, Experiment 2 only required simple color judgments before making a motor response, while Experiment 3 and 4 involved very simple signal-response tasks with minimal cognitive processing. Therefore, we speculate that whether mind wandering slows down reaction times depends on the need for complex cognitive processing before the motor response. Understanding how mind wandering influences reaction times across different task types is crucial, but it was not the primary focus of the current study. We hope that future research can systematically explore this issue in greater depth.

Supplementary Figures

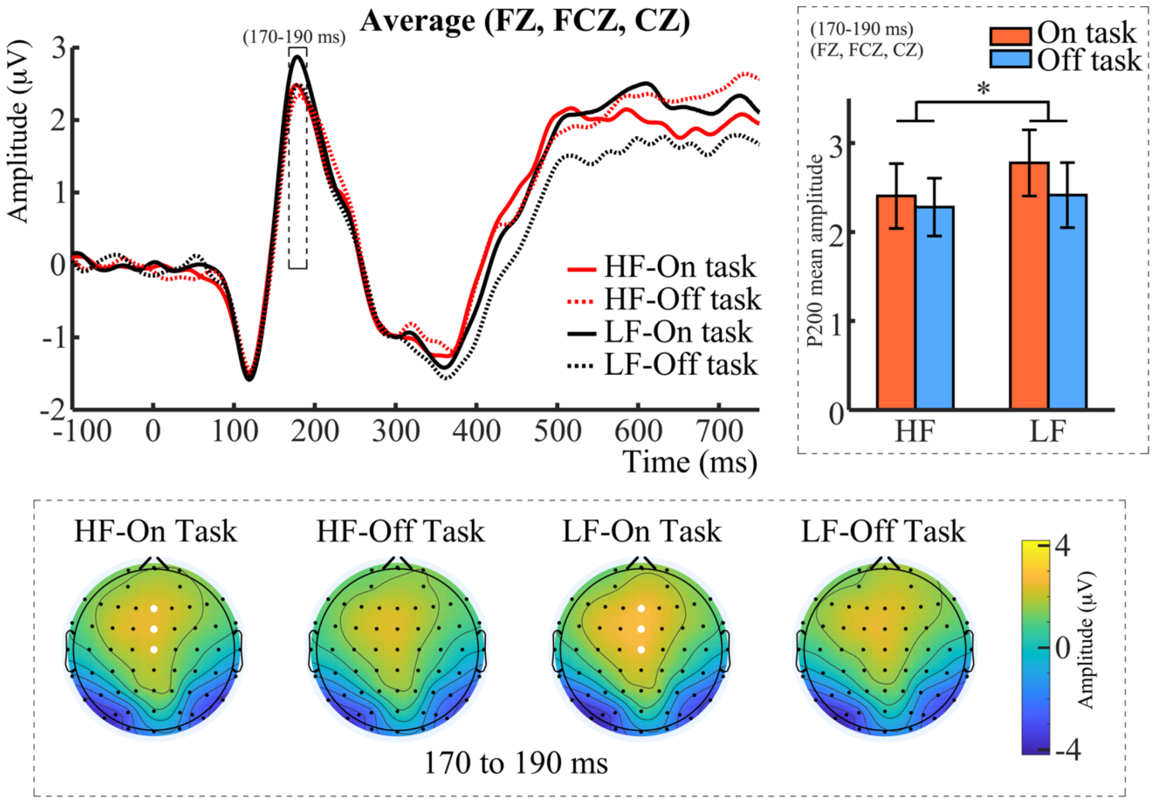

Fig. S1. Results of P200 component in Experiment 1. In the literature, the P200 component is usually associated with early phonological or orthographic activation in visual word recognition (19–21). Furthermore, Sereno et al. (20) and Barnea and Breznitz (19) suggested that the P200 component may indicate vocabulary processing and can be modulated by factors such as lexicality, word frequency, and word regularity. Wang et al. (22) have found that low-frequency two-character compound Chinese words elicited a greater P200 component than high-frequency words, which indicates that semantic information plays an important role during phonological access. In Experiment 1 of this study, the peak of the P200 component occurred at 180 ms. We defined a time window for the P200 centered around this peak, spanning 20 ms from 170 to 190 ms (left upper panel). Within this 170-190 ms time window, the P200 was predominantly distributed over midline electrode sites on the scalp (lower panel). We selected FZ, FCZ, and CZ (marked by white dots on the scalp topography) as representative electrodes for the P200, averaged the amplitudes across these three electrodes, and then conducted a 2 (Word Frequency: high vs. low) × 2 (Probe: on task vs. off task) repeated-measures ANOVA (right upper panel). It revealed a significant main effect of Word Frequency, $F(1, 28) = 4.68$, $p = 0.039$, $\eta_p^2 = 0.143$, a marginal main effect of Probe, $F(1, 28) = 3.62$, $p = 0.067$, $\eta_p^2 = 0.115$, but no significant interaction between Word Frequency and Probe, $F(1, 28) = 1.77$, $p = 0.19$, $\eta_p^2 = 0.059$. Since semantic information plays a role in phonological access, our results revealed that low-frequency words elicited a larger P200 component compared to high-frequency words. This indicates that the semantic processing difficulty of low-frequency words in Experiment 1 is indeed greater than that of high-frequency words, further supporting the effectiveness of manipulating task difficulty through word frequency.

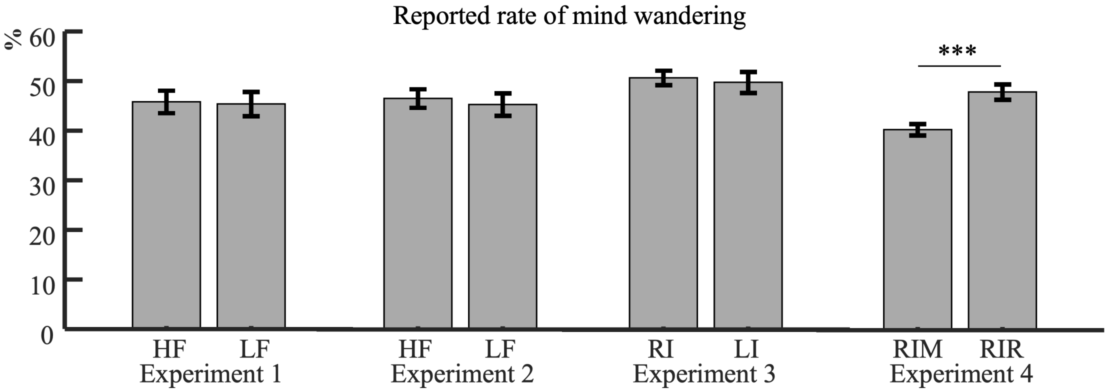

Fig. S2. The reported rate of mind wandering across Experiments 1 to 4. The goal of many experimental designs in the four EEG experiments of the current study was to achieve a mind wandering report rate close to 50%. Our results support the validity of those designs. In Experiment 1, the average proportion of mind wandering reports in the high-frequency word condition (M = 45.79%, SE = 2.27%) did not differ significantly from that in the low-frequency word condition (M = 45.36%, SE = 2.46%), $t(28) = 0.220$, $p = 0.827$, Cohen's $d = 0.041$. In Experiment 2, there was no significant difference in the average proportion of mind-wandering reports between the high-frequency (M = 46.55%, SE = 1.88%) and low-frequency (M = 45.31%, SE = 2.27%) word conditions, $t(28) = 0.377$, $p = 0.709$, Cohen's $d = 0.07$. In Experiment 3, the average proportion of mind wandering reports in the right-hand (RI) condition (M = 50.73%, SE = 1.46%) did not differ significantly from that in the left-hand (LI) condition (M = 49.83%, SE = 2.12%), $t(27) = 0.433$, p = 0.668, Cohen's $d = 0.082$. In Experiment 4, the average proportion of mind wandering reports in the key-releasing with RIM condition (M = 40.38%, SE = 1.16%) was significantly lower than that in the key-releasing with RIR condition (M = 47.97%, SE = 1.54%), $t(28) = -3.968$, $p < 0.001$, Cohen's $d = 0.737$. Overall, these results differ from our expectations. We hypothesized that easier task conditions would leave more executive control resources available for mind wandering, leading to a higher proportion of mind wandering reports. However, in Experiments 1 and 3, we did not find a higher proportion of mind wandering reports in the easier task conditions (i.e., HF and RI) compared to the more difficult task conditions (i.e., LF and LI). We speculate that this discrepancy may be due to the block design of our experiments, where the easier and more difficult task conditions alternated throughout the experiment. On the timescale of each block, the task difficulty varies, but on the timescale of the whole experiment, the two levels of difficulty are mixed (since the two types of blocks alternate). As a result, the effect of task difficulty on the overall proportion of mind wandering reports may become less distinct. In other words, if the easier and more difficult tasks are conducted separately, the mind wandering report rate in the easier tasks would be higher than in the more difficult ones.

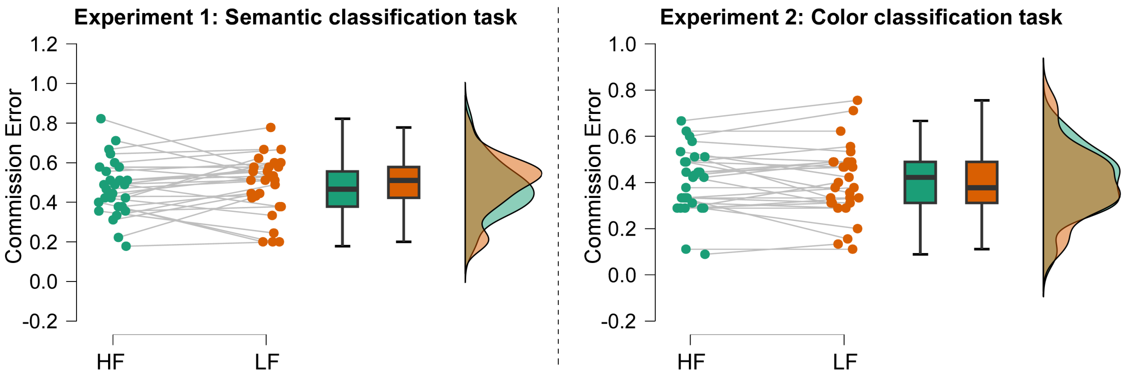

Fig. S3. The commission errors rate between high- and low-frequency words across Experiments 1 to 2. Commission errors in the Sustained Attention to Response Task (SART) occur when participants incorrectly press a response key during NOGO trials (e.g., the "animal" words in Experiment 1). In the current study, we employed the SART paradigm with Chinese word stimuli. Critically, we manipulated task difficulty operationally through word frequency, where low-frequency words elicited slower response times (RTs) compared to high-frequency words, as anticipated (Experiment 1). However, despite the marked difference in RTs between high- and low-frequency conditions, no significant differences in commission error rates were observed between these groups in either experiment (Experiment 1: $t(28) = -0.629$, $p = 0.534$, Cohen's $d = -0.117$; Experiment 2: $t(28) = 0.446$, $p = 0.659$, Cohen's $d = 0.083$). This finding is critical because it suggests that while task difficulty (as indexed by RTs) differed between conditions, the fundamental speed–accuracy trade-off dynamic inherent to the SART paradigm remained intact. As noted by Seli et al. (23), commission errors in the SART are traditionally interpreted as failures of sustained attention but can also reflect procedural artifacts arising from speed-prioritized responding. In their study, explicit instructions to prioritize accuracy over speed reduced commission errors by minimizing impulsive responses, indicating that speed–accuracy trade-offs are highly sensitive to task instructions. Crucially, in our experiments, even though word frequency induced variability in RTs (with low-frequency words requiring slower responses), the lack of divergence in commission errors between conditions suggests that the attentional demands of withholding responses to NOGO stimuli were not conflated with task difficulty or RT variability. This aligns with the findings of Seli et al. (23), who demonstrated that strategic adjustments in responding (e.g., slowing RTs) do not inherently disrupt the SART's capacity to isolate attention lapses, provided accuracy remains a stable metric.

Furthermore, our results align with the observation that commission errors are driven by U-shaped relationships with RTs, where both excessively fast and slow responses correlate with errors (23). In our paradigm, even though low-frequency words elicited slower RTs overall, the critical NOGO trials were not systematically influenced by this RT slowing, as evidenced by comparable error rates across conditions. This indicates that RT modulation induced by word frequency did not shift participants outside the "Goldilocks zone" (300–800 ms), where optimal inhibitory control is exercised. Thus, our use of word frequency to manipulate task difficulty preserved the integrity of the SART's attentional metrics, as neither speed adjustments nor task difficulty introduced confounds to the core speed–accuracy trade-off.

In conclusion, the absence of commission error differences between high- and low-frequency word conditions, despite RT disparities, demonstrates that our operationalization of task difficulty through word frequency is methodologically valid within the SART framework.

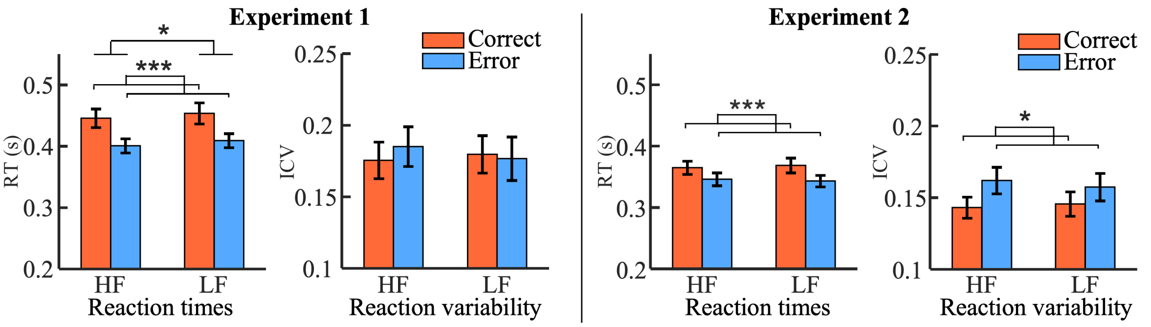

Fig. S4. The reaction times and reaction variability for the trials preceding target stimuli across Experiments 1 to 2. Experiments 1 and 2 utilized the SART paradigm, allowing for the analysis not only of trials preceding the thought probes but also of those preceding target stimuli. If participants correctly withheld their responses to the target, data from the six nontarget trials preceding the target were categorized as "correct" trials. Otherwise, they were categorized as "error" trials. These erroneous responses are referred to as "commission errors," which some studies suggest representing an objective behavioral index of mind wandering (24, 25). For the behavioral reaction times and reaction variability (ICV) in the six trials preceding the targets in Experiments 1 and 2, we conducted a 2 (target: Correct vs. Error) × 2 (word frequency: HF vs. LF) repeated-measures ANOVA. For the reaction times in Experiment 1, it revealed a significant main effect of target, $F(1, 28) = 27.389$, $p < 0.001$, $\eta_p^2 = 0.494$, a significant main effect of word frequency, $F(1, 28) = 4.595$, $p = 0.041$, $\eta_p^2 = 0.141$, but no significant interaction between target and word frequency, $F(1, 28) = 0.004$, $p = 0.952$, $\eta_p^2 < 0.001$. This result indicates that when participants were required to respond to word semantics, reaction times were significantly slower for low-frequency words compared to high-frequency words. Additionally, during commission errors, reaction times were faster than during correct responses to targets, suggesting that participants exhibited more impulsive responses. For the reaction variability in Experiment 1, it revealed no significant main effect of word frequency, $F(1, 28) = 0.254$, $p = 0.618$, $\eta_p^2 = 0.009$, no significant main effect of target, $F(1, 28) = 0.170$, $p = 0.684$, $\eta_p^2 = 0.006$, and no significant interaction between target and word frequency, $F(1, 28) = 1.812$, $p = 0.189$, $\eta_p^2 = 0.061$.

For the reaction times in Experiment 2, it revealed a significant main effect of target, $F(1, 28) = 20.607$, $p < 0.001$, $\eta_p^2 = 0.424$, but no significant main effect of word frequency, $F(1, 28) = 0.033$, $p = 0.857$, $\eta_p^2 = 0.001$, no significant interaction between target and word frequency, $F(1, 28) = 1.598$, $p = 0.217$, $\eta_p^2 = 0.054$. This result indicates that when participants were required to respond to color, there was no significant difference in reaction times between low-frequency and high-frequency words. Similar to Experiment 1, reaction times during commission errors were faster than those during correct responses to targets. For the reaction variability in Experiment 2, it revealed a significant main effect of target, $F(1, 28) = 6.719$, $p = 0.015$, $\eta_p^2 = 0.194$, but no significant main effect of word frequency, $F(1, 28) = 0.036$, $p = 0.852$, $\eta_p^2 = 0.001$, no significant interaction between target and word frequency, $F(1, 28) = 0.946$, $p = 0.339$, $\eta_p^2 = 0.033$. This result indicates that reaction variability was significantly greater for "error" trials than for "correct" trials.

Although previous studies have proposed that commission errors may reflect mind wandering, our findings showed that commission errors, unlike off-task reports, did not consistently result in increased reaction time variability in different task context. Instead, commission errors were associated with faster reaction times, indicating more impulsive behavioral responses. This supports prior research suggesting that commission errors reflect motor decoupling rather than attention lapses (2, 26). Our results, at least within the context of the current study, suggest that commission errors and off-task reports represent distinct cognitive processes.

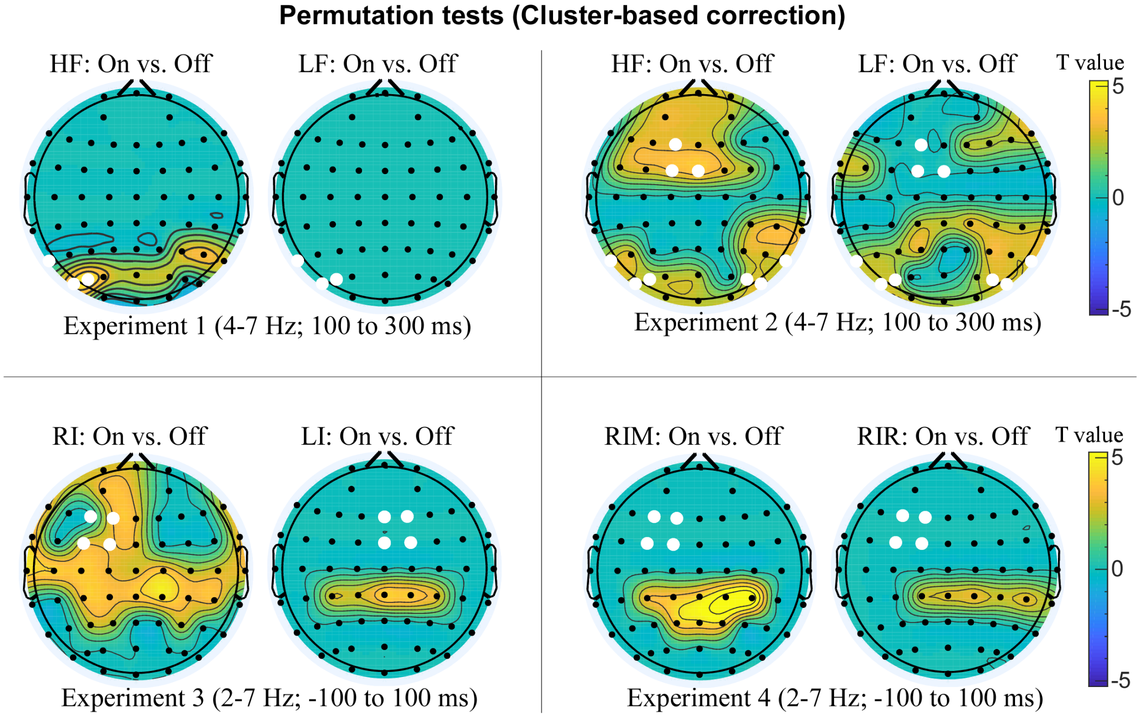

Fig. S5. The electrode selection for ITPC statistical analyses was supported by permutation tests. In the current study, we focused on inter-trial phase coherence (ITPC) within specific frequency bands and time windows based on prior knowledge. For Experiments 1 and 2, we analyzed the 4–7 Hz band (100–300 ms), while Experiments 3 and 4 examined the 2–7 Hz band (−100–100 ms). Although electrode selections were guided by existing literature, we further validated their validity by presenting results across all electrodes, ensuring that our chosen sites accurately reflected the neural activity of interest. Cluster-based permutation tests (corrected for multiple comparisons) were employed to evaluate the robustness of these selections. All four experiments adopted a 2×2 within-subject repeated-measures design, with a particular emphasis on task difficulty as a key moderator. However, despite our hypothesis, permutation tests failed to reveal significant interaction effects in Experiment 1 and 3. Critically, our primary analyses relied on a priori-defined ITPC parameters (specific frequency bands, time windows, and electrodes) through repeated-measures ANOVAs, independent of permutation outcomes. The permutation results served only as auxiliary evidence to confirm the rationality of electrode selection. For each experiment, two cluster-corrected permutation comparisons were conducted (e.g., in Experiment 1: HF-On vs. HF-Off and LF-On vs. LF-Off tasks), using a Monte Carlo approach with 1000 permutations per contrast. At each iteration, paired-sample t-tests were computed across participants for all time-frequency-electrode combinations. Cluster-level statistics were derived by thresholding suprathreshold electrode/time points ($p < 0.05$, two-tailed) and merging spatially/temporally contiguous clusters, with the final cluster p-values adjusted for family-wise error using the permutation distribution of maximum cluster masses. This approach controlled for multiple comparisons while preserving spatiotemporal specificity of neural effects. Significant electrode clusters were identified in the 4–7 Hz (100–300 ms) range for Experiments 1 and in the 2–7 Hz (−100–100 ms) range for Experiments 3–4. These results collectively affirm that the electrodes reported in the main text are representative of the neural synchronization patterns under investigation. (The main electrodes used for repeated-measures ANOVAs in the main text are highlighted in white within the figures; T-values of electrodes lacking statistical significance are set to zero to enhance the visualization of significant electrode clusters).

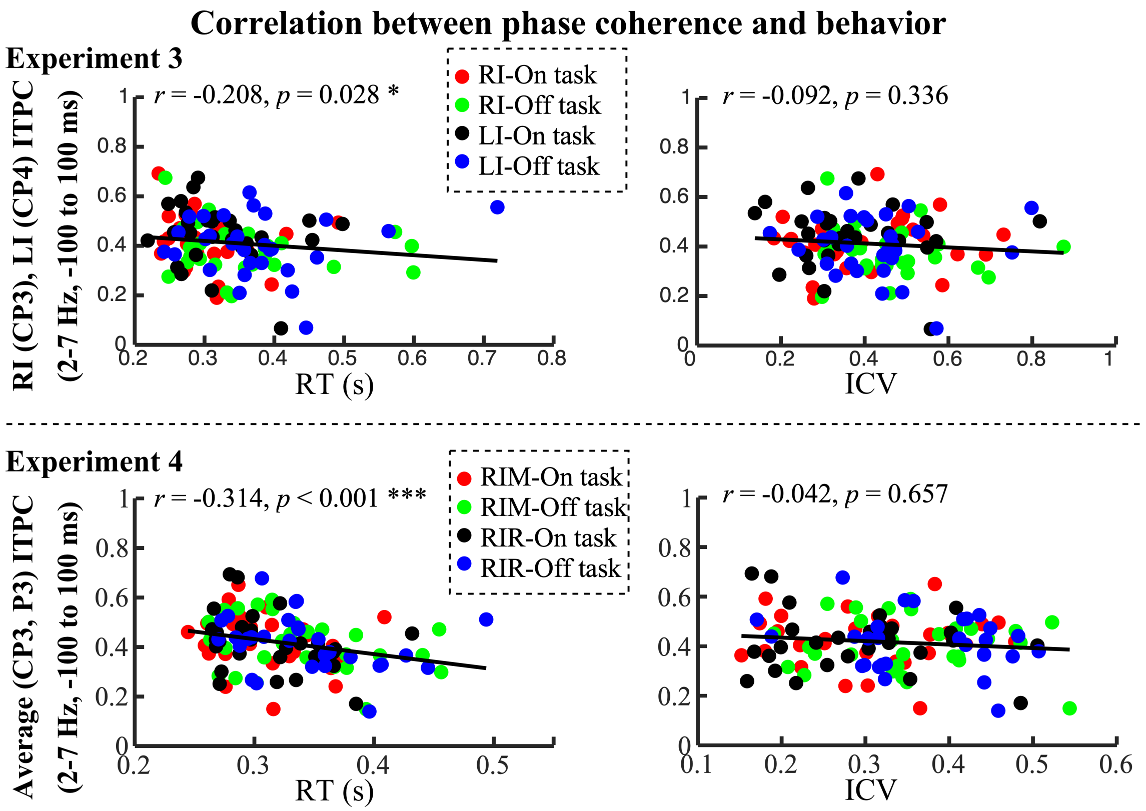

Fig. S6. Correlation between phase coherence and behavioral responses in Experiment 3 and 4. During the motor tasks in Experiments 3 and 4, significant overall negative correlations were observed only at the centro-parietal electrodes, with no correlation found for reaction variability. Spearman's correlation coefficient was used to assess these relationships.

SI References

1. F. Faul, E. Erdfelder, A. Buchner, A.-G. Lang, Statistical Power Analyses Using G*Power 3.1: Tests for Correlation and Regression Analyses. *Behavior Research Methods* **41**, 1149–1160 (2009).
2. Z. Long, Q. Fu, X. Fu, Word Familiarity Modulates the Interference Effects of Mind Wandering on Semantic and Reafferent Information Processing. *Journal of cognitive neuroscience* **35**, 1229–1245 (2023).
3. J. Xu, D. Friedman, J. Metcalfe, Attenuation of deep semantic processing during mind wandering. *NeuroReport* **29**, 380–384 (2018).
4. P. Seli, M. Konishi, E. F. Risko, D. Smilek, The role of task difficulty in theoretical accounts of mind wandering. *Consciousness and Cognition* **65**, 255–262 (2018).
5. P. Seli, J. S. A. Carriere, M. Levene, D. Smilek, How few and far between? Examining the effects of probe rate on self-reported mind wandering. *Frontiers in Psychology* **4** (2013).
6. Z. Long, Q. Fu, X. Fu, How mind wandering influences motor control: the modulating role of movement difficulty. *NeuroImage* **294**, 120638 (2024).
7. A. Delorme, S. Makeig, EEGLAB: an open source toolbox for analysis of single-trial EEG dynamics including independent component analysis. *Journal of Neuroscience Methods* **134**, 9–21 (2004).
8. J. Rodrigues, M. Weiß, J. Hewig, J. J. B. Allen, EPOS: EEG Processing Open-Source Scripts. *Frontiers in Neuroscience* **15** (2021).
9. M. X. Cohen, *Analyzing neural time series data : theory and practice* (The Mit Press, 2014).
10. R. M. van Diepen, A. Mazaheri, The Caveats of observing Inter-Trial Phase-Coherence in Cognitive Neuroscience. *Scientific Reports* **8** (2018).
11. A. Cheviet, A. Bonnefond, F. Bertrand, M. Maumy-Bertrand, N. Doignon-Camus, How visual attention span and phonological skills contribute to N170 print tuning: An EEG study in French dyslexic students. *Brain and Language* **234**, 105176 (2022).
12. S. Dehaene, L. Cohen, The unique role of the visual word form area in reading. *Trends in Cognitive Sciences* **15**, 254–262 (2011).
13. Y. Pu, D. Cheyne, Y. Sun, B. W. Johnson, Theta oscillations support the interface between language and memory. *NeuroImage* **215**, 116782 (2020).
14. S. J. Luck, *An introduction to the event-related potential technique*, 2nd Ed. (MIT Press, 2014).
15. J. F. Cavanagh, M. J. Frank, Frontal theta as a mechanism for cognitive control. *Trends in Cognitive Sciences* **18**, 414–421 (2014).
16. T. Womelsdorf, M. Vinck, L. S. Leung, S. Everling, Selective Theta-Synchronization of Choice-Relevant Information Subserves Goal-Directed Behavior. *Frontiers in Human Neuroscience* **4**, 210 (2010).
17. C.-H. Wang, C.-T. Yang, D. Moreau, N. G. Muggleton, Motor expertise modulates neural oscillations and temporal dynamics of cognitive control. *NeuroImage* **158**, 260–270 (2017).
18. S. Popovych, *et al.*, Movement-related phase locking in the delta–theta frequency band. *NeuroImage* **139**, 439–449 (2016).
19. A. Barnea, Z. Breznitz, Phonological and Orthographic Processing of Hebrew Words: Electrophysiological Aspects. *The Journal of Genetic Psychology* **159**, 492–504 (1998).
20. S. C. Sereno, K. Rayner, M. I. Posner, Establishing a time-line of word recognition: evidence from eye movements and event-related potentials. *NeuroReport* **9**, 2195–2200 (1998).
21. B. Liu, Z. Jin, Z. Qing, Z. Wang, The processing of phonological, orthographical, and lexical information of Chinese characters in sentence contexts: An ERP study. *Brain Research* **1372**, 81–91 (2011).
22. Y. Wang, M. Jiang, Y. Huang, P. Qiu, An ERP Study on the Role of Phonological Processing in Reading Two-Character Compound Chinese Words of High and Low Frequency. *Frontiers in Psychology* **12** (2021).
23. P. Seli, J. A. Cheyne, D. Smilek, Attention failures versus misplaced diligence: Separating

attention lapses from speed–accuracy trade-offs. *Consciousness and Cognition* **21**, 277–291 (2012).
24. D. Smilek, J. S. A. Carriere, J. A. Cheyne, Failures of sustained attention in life, lab, and brain: Ecological validity of the SART. *Neuropsychologia* **48**, 2564–2570 (2010).
25. J. A. Cheyne, J. S. A. Carriere, D. Smilek, Absent-mindedness: Lapses of conscious awareness and everyday cognitive failures. *Consciousness and Cognition* **15**, 578–592 (2006).
26. J. Head, W. S. Helton, Perceptual decoupling or motor decoupling? *Consciousness and Cognition* **22**, 913–919 (2013).
